# Machine learning-based virtual screening and density functional theory characterisation of natural inhibitors targeting mutant PBP2x in *Streptococcus pneumoniae*

**DOI:** 10.1038/s41598-025-24222-1

**Published:** 2025-11-07

**Authors:** Avani Panickar, Anand Manoharan, Sudha Ramaiah

**Affiliations:** 1 Medical and Biological Computing Laboratory, School of Biosciences and Technology, Vellore Institute of Technology (VIT), Vellore, Tamil Nadu 632014 India; 2 Department of Bio-Sciences, School of Biosciences and Technology, Vellore Institute of Technology (VIT), Vellore, Tamil Nadu 632014 India; 3 Infectious Diseases Medical and Scientific Affairs, GlaxoSmithKline (GSK), Worli, Maharashtra 400030 India

**Keywords:** Glucozaluzanin c, Penicillin binding protein 2x, Molecular dynamic simulation, Molecular docking, Elephantopus scaber, Β-lactam-resistant, Computational models, Machine learning, Predictive medicine, Virtual drug screening, Drug discovery, Plant sciences, Structural biology, Microbiology, Bacteria, Pathogens

## Abstract

*Streptococcus pneumoniae* (*S. pneumoniae*) has developed resistance to β-lactam antibiotics, largely due to mutations in penicillin-binding protein 2x (PBP2x), particularly within conserved motifs such as STMK and KSG. PBP2x mutations are frequently reported in multidrug-resistant pneumococcal strains associated with pneumonia, meningitis, and septicaemia. especially in serotypes 19A, 19F, and 23F, showing reduced susceptibility to β-lactam antibiotics. These mutations in the PBP2x disrupt antibiotic binding and enzymatic functions, highlighting the need for alternative therapeutic strategies. This study focused on five clinically relevant PBP2x mutations (T338A/G/P and K547G/T) within its active site. A library of phytocompounds was screened using a machine learning model trained to identify antibacterial compounds. Top candidates were filtered based on ADMET properties, and their electronic characteristics were assessed using HOMO–LUMO analysis and electrostatic potential mapping, through density functional theory (DFT). Glucozaluzanin C, a phytochemical derived from *Elephantopus scaber*, emerged as a potential candidate. Molecular docking and dynamics simulations revealed strong binding affinity and structural integrity with all PBP2x mutants, over a 100-ns timescale. RMSD, RMSF, and hydrogen bonding analysis confirmed stable interactions, suggesting Glucozaluzanin C may effectively interact with PBP2x mutants. Overall, the study highlights an effective strategy for identifying plant-derived inhibitors against β-lactam-resistant *S. pneumoniae.*

**Supplementary Information:**

The online version contains supplementary material available at 10.1038/s41598-025-24222-1.

## Introduction

The prevalence of multidrug-resistant *Streptococcus pneumoniae* (*S. pneumoniae*) has been increasing since it was first reported in the 1960s^1^. *Streptococcus pneumoniae*, a clinically important pathogen, causes a broad spectrum of invasive and non-invasive infections such as pneumonia, meningitis, septicaemia, and otitis media. It remains a major contributor to morbidity and mortality, particularly among children under five years of age, adults over 60 years, and immunocompromised individuals. Although the widespread administration of β-lactam antibiotics has significantly contributed to the management of pneumococcal infections for decades, the emergence of resistance to these antibiotics has become a global concern. In some countries, *S. pneumoniae* strains now show reduced susceptibility to broad β-lactams such as amoxicillin^2,3^. The mechanism of resistance in *S. pneumoniae* is largely recognised by the structural modification in penicillin-binding proteins (PBPs), which serve as the main targets of β-lactams. *S. pneumoniae* genome encodes six PBPs, and among these, PBP2x plays a major role in peptidoglycan cross-linking and cell wall biosynthesis and is one of the first PBPs to acquire mutations under antibiotic pressure^4^. Mutations in the PBP2x protein can result in low-affinity PBP2x variants, reducing the efficacy of the β-lactam antibiotics and contributing to antimicrobial resistance (AMR)^5^. Specific amino acid substitutions in the transpeptidase domain of PBP2x, such as T338A/G/P and K457G/T, have been identified for causing reduced β-lactam binding^6^. These mutations alter the geometric charge distribution of the active site, weakening the interaction between the antibiotic and the serine residue required for acylation and inhibition^7,8^. In clinical isolates, mutations are often associated with a high level of resistance, making traditional antibiotics ineffective. The T338 residue, part of the SXXK motif, plays a crucial role in forming hydrogen bond networks and stabilising substrate interactions. Alteration at this site leads to an “open” active site configuration, disrupting water-mediated interactions essential for drug binding^9^. Similarly, mutations at K547 near the catalytic groove affect the electrostatic interactions and substrate stabilisation, decreasing drug effectiveness. Understanding these structural changes highlights the importance of focusing on specific PBP2x mutations when designing more effective, targeted inhibitors^10^. Our previous work^11^ identified high-frequency PBP2x mutations in clinical pneumococcal isolates recovered from patients with pneumonia, meningitis, and septicaemia in India and Vietnam. Similar patterns have been reported in China, South Korea, and Japan, particularly in multidrug-resistant serotypes like 19A, 19F, 6B, and 23F. These serotypes are commonly associated with both community-acquired pneumonia and invasive pneumococcal diseases and are frequently resistant to β-lactams. The regional spread and resistance profile underscore the importance of developing new antimicrobial inventions targeting conserved mutations in PBP2x^12^.

As β-lactam antibiotics face increasing limitations against resistant PBP2x variants, the investigation for novel therapeutic strategies has been directed towards plant-driven natural compounds. Phytochemicals such as phenolics, flavonoids, and terpenoids exhibit diverse chemical scaffolds and have demonstrated intrinsic antibacterial properties. Some are known to bind and inhibit PBPs allosterically^13,14^. Recent in silico studies have reported promising interactions of phytochemicals such as silicritin, gallocatechin gallate, and curcumin analogues with PBP2x, suggesting their potential role in overcoming resistance mechanisms^8,15^. Based on this evidence, Machine learning (ML) algorithms analyse large-scale molecular datasets to identify patterns in physicochemical properties and predict the possibility of strong binding to specific protein targets. By combining ML-driven prediction models with high-throughput virtual screening, it is possible to prioritise phytocompounds that exhibit favourable interaction profiles^16,17^.

**Fig. 1:**
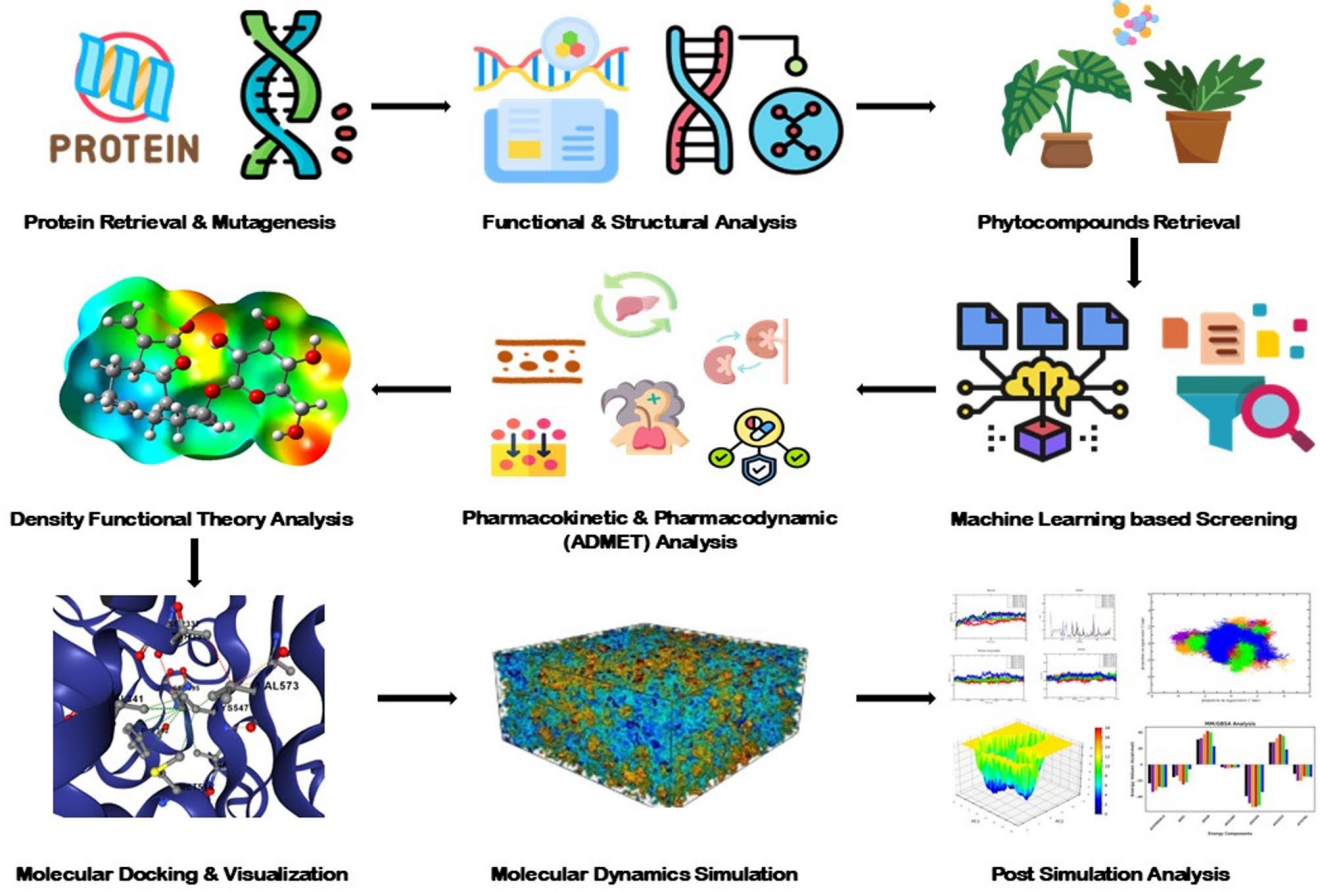
Schematic workflow of machine learning-based virtual screening and DFT characterisation of natural inhibitors targeting mutant PBP2x in *S. pneumoniae*.

The present study builds on our previous work and aims to identify plant-derived inhibitors targeting mutant forms of PBP2x in *S. pneumoniae*, specifically those with T338A/G/P and K547G/T substitutions associated with β-lactam resistance. Accordingly, we utilised a screening approach that integrates ML-based compound screening, density functional theory (DFT) analysis for reactivity validation, and molecular docking simulations to evaluate binding interactions as represented in Fig. 1. By focusing on structurally characterised resistance mutations, this study aims to discover phytocompounds with high binding affinity and structural stability, contributing to the development of targeted therapeutic options against β-lactam-resistant *S. pneumoniae.*

## Methods

### Protein retrieval and mutant generation

The reference sequence of the PBP2x protein (Accession ID: COS99621) from *S. pneumoniae* (GenBank Accession: LN831051) was retrieved from the NCBI database. A BLASTp search against the Protein Data Bank (PDB) was then conducted to identify a suitable 3D crystal structure. The structure with the highest sequence similarity (> 99%) and maximum alignment coverage was chosen, specifically the crystal structure of PBP2x (PDB ID: 1QME). The selected structure was visualised using PyMOL software, and all heteroatoms, including water molecules and ligands, were removed to prepare the protein for subsequent analysis. Following structural preparation, site-directed mutagenesis was carried out using the SPDB Viewer to introduce clinically relevant amino acid substitutions. The mutations T338A/G/P within the conserved SXXK (STMK) motif, and K547G/T adjacent to the KXG (KSG) motif were introduced. These mutations have been previously associated with reduced susceptibility to amoxicillin and are commonly observed in multidrug-resistant clinical isolates^18,19^. The prepared wild-type and mutant protein models were then used for further analysis^20^.

### Evaluation of functional and structural impact of mutations

To assess the functional impact of amino acid substitutions in the PBP2x protein, we used PROVEAN and Meta-SNP. PROVEAN predicts whether an amino acid substitution or indel has a neutral or deleterious based on sequence homology and alignment-based scoring^21^. Meta-SNP, a consensus classifier integrating SIFT, PhD-SNP, SNAP, and PANTHER, was used for cross-validation to determine single-nucleotide polymorphism SNP pathogenicity^22^. We also applied ConSurf for identifying the evolutionarily conserved region within the PBP2x^23^. Additionally, we used DynaMut2 to evaluate the impact of mutations on protein stability. It combines normal mode analysis and graph-based features to estimate ΔΔG values, indicating stabilizing and destabilizing effects^24^.

### Phytocompound curation and model prediction

#### Data preparation

Bioassay data for the compounds active against *S. pneumoniae* were retrieved from the PubChem Bioassay database (AID 438298), comprising 36 active and 5 inactive compounds. To improve classification performance and address class imbalance, additional inactive compounds were included. These curated compounds formed the core dataset for model development. For external testing, structurally complete phytocompounds with traditional use against pneumonia were selected from the IMPPAT (Indian Medicinal Plants, Phytochemistry and Therapeutics) database^25^.

#### Descriptor calculations

All compounds were converted to 3D-Standard Format (3D-SDF), and molecular descriptors were generated using PaDEL-Descriptor^26,27^. This included 1D, 2D, and 3D features along with chemical fingerprints such as MACCS keys and PubChem substructures. Feature selection was carried out using WEKA (Waikato Environment for Knowledge Analysis) bult-in evaluation methods to retain only the most informative descriptors for model training^28,29^.

#### Machine-learning model prediction

Supervised learning was performed using tenfold cross-validation^30^. Classifiers including Random Forest, J48, PART, Reduced Pruning Tree (RepTree) were tested within WEKA, chosen for their proven reliability in virtual screening applications^31,32^. Model performance was evaluated using standard matrices such as accuracy, precision, sensitivity, specificity, F1-score, and area under the ROC curve (AUC)^33^. Confusion matrices were generated to assess classification outcomes and ensure robustness across models.

### ADMET screening evaluation

The ADMET (absorption, distribution, metabolism, excretion, and Toxicity) profiling was carried out to evaluate the pharmacokinetic properties and safety of the selected phytocompounds, which are essential for assessing drug-likeness^34^. The analysis used ADMETlab 3.0, a web-based tool that predicts multiple ADMET parameters that is widely used in virtual screening workflows for both natural and synthetic compounds^35,36^. Toxicity was assessed using ProTox 3.0, which predicts endpoints such as LD50, hepatotoxicity, and carcinogenicity based on structure-activity relationships and machine learning models^37,38^. The combined use of ADMETlab and ProTox enables efficient screening of the candidate phytocompounds and eliminates those with poor pharmacokinetic and safety profiles. Compounds showing favourable ADME properties and minimal toxicity were selected and used for further analysis.

### Density functionality theory (DFT) analysis

The stability and reactivity of selected phytocompounds were evaluated using DFT, a quantum chemistry method widely employed to examine molecular structures, electronic properties, and reactivity^39^. Geometry optimisations were performed using the B3LP functional with the 6–311g++ (d, p) basis set in Gaussian09W^40^. structural visualisation was carried out in GuassView 6.0. To assess molecular reactivity, frontier molecular orbitals (FMOs) [Highest Occupied Molecular Orbital (HOMO) and Lowest Unoccupied Molecular Orbitals (LUMO)], were analysed. Key reactivity descriptors such as ionisation potential, electron affinity, chemical hardness, and electrophilicity were calculated based on Koopmans theorem. Electrostatic potential (ESP) maps were generated to visualise electron-rich and electron-deficient regions, aiding the identification of likely nucleophilic or electrophilic attack sites^41^. Frequency calculations confirmed that the optimised geometries corresponded to true energy minima (no imaginary frequencies). Additionally, IR and Raman spectra were simulated to support structural characterisation^42^.

### Protein-ligand interactions analysis

Molecular docking analyses were conducted to explore the interactions between the selected compound and the wildtype as well as mutant forms of PBP2x. Docking was performed using AutoDock v1.5.7, a widely used and validated platform for structure-based drug design^43,44^. Both the wild type and mutant structures of PBP2x were prepared for docking by introducing polar hydrogen atoms, Kollam unit atom charges and merging non-polar hydrogen atoms. This preparation step stabilises the finishing point and ensures accurate representation of the protein’s electrostatic surface^45^. Ligands were prepared by fixing torsions and assigning Gasteiger charges, allowing for precise modelling of conformational flexibility and charge distribution^46^. The active site of PBP2x, including mutation hotspots (T338A/G/P and K547G/T), was visualised using PyMOL 2.5. Coordinates for the docking grid were extracted based on the known binding pocket, centred at X = 110.427, Y = 62.793, and Z = 74.090, with grid dimensions of 60 × 60 Å^3^ and a spacing of 0.375 Å, ensuring complete coverage of the active site^47^. Docking analyses were performed using the Lamarckian Genetic Algorithm, which incorporates evolutionary search with local optimisation for efficient conformation generation. The lowest binding energy (BE) docked structure was selected for each protein-ligand pair and further analysed^48^. The best docked complexes were visualised and analysed using BIOVIA Discovery Studio Visualizer 2020^49^. The best complex was further used for the Molecular Dynamics (MD) simulation.

### Stability assessments of Protein-Ligand complex

MD simulations were carried out using GROMACS v2024.2 ^50^, to evaluate the stability and dynamics of the protein-ligand complexes. Docked complexes involving the PBP2x wild-type and mutant forms were selected, along with the apo form of the protein for comparative analysis^51^. System topologies were generated using the CHARMM general field of force (CGennFF)^52^, which provides parameters suitable for drug-like molecules. Each system was placed in a dodecahedral simulation box with a minimum 1.0 nm distance from the solute and solvated using TIP3P water molecules^53^. Counter-ions were added to neutralise the system and replicate physiological ionic strength. Energy minimisation was performed using the steepest descent algorithm for 50,000 steps, with a force convergence criterion of 10 kJ/mol. The Verlet cutoff scheme was used to handle non-bonded interactions^54^. Equilibration was carried out in two phases: first under the NVT ensemble (100 ps) to stabilise temperature, followed by NPT equilibration (100 ps) to stabilise pressure, both at 310 K and 1 bar. The velocity-rescale thermostat was used for temperature coupling, and the Parrinello-Rahman barostat was applied for pressure coupling^55,56^. A 100 ns production MD run was conducted with periodic boundary conditions and a 2 fs integration time step. Electrostatic interactions were treated using the Particle Mesh Ewald (PME) method, and all bond lengths involving hydrogen atoms were constrained using the LINCS algorithm.

Trajectory analyses were performed to evaluate structural stability and motions. Principal Component Analysis (PCA) was used to identify major conformational changes based on the backbone atom fluctuations^57^. The covariance matrix was calculated using gmx covar, and eigenvectors were extracted via gmx anaeig. The first two principal components (PC1 and PC2), representing dominant motions^58,59^, were used to generate Free Energy Landscape (FEL) plots. FELs were constructed using a Python script that computed energy basins and conformer stability across the simulation landscape^60^. Biding free energy calculations were performed using the Molecular Mechanics/ Generalised Born Surface Area (MM/GBSA) incorporated in gmx_MMPBSA program. A total of 10,000 frames were extracted from the 100ns trajectory^61^. The solvation model parameter (igb) was set to 5, with internal and external dielectric constants of 1.0 and 78.5, respectively, to simulate polarisation effects^62^. In addition, per-residue decomposition analysis was performed to quantify individual amino acid contributions to the overall binding energy, identifying key interacting residues across complexes^63,64^.

## Result

### Mutant and structure generation

The mutations selected for this study were identified through a comprehensive literature review, which highlighted clinically relevant mutants in the PBP2x, a region critical for β-Lactam antibiotic binding. Specifically, we focused on two key active-site motifs, mainly SXXK and KSG. Mutations at positions T338A/G/P (within the STMK motif) and K547G/T (within the KSG motif) were targeted. These mutations were introduced into the wild-type PBP2x structure retrieved from the PDB. A total of five mutations (T338A, T338G, T338P, K547G, K547T) were induced into the structure for further analysis. Notably, both mutation sites are located within the same binding pocket.

### Mutation impact analysis

The functional impact of amino acid substitutions in the PBP2x protein was assessed using SNP tools showed that all five selected mutations that are STMK (T338A, T338G, T338P) and KSG (K547G, K547T) were predicted to be deleterious (Supplementary Table S1). Conservation analysis indicated that these residues are highly conserved and are positioned in functionally important regions of the protein. Surface accessibility mapping further showed that these residues are surface-exposed. Protein stability changes upon mutation were evaluated, which calculates ∆∆G values based on normal model analysis. All five mutations showed negative ∆∆G values, indicating a destabilising effect on protein structure. The extent of destabilisation varied among the mutations, as summarised in Supplementary Table S2. The predicted structural change for the protein in the Supplementary Figs. S1 and S2.

### ML-Based screening of phytocompounds

A total of 10,000 phytocompounds were initially collected from 111 different plant species known for their activity against bacterial pneumonia. After screening and removing all the duplicate entries, a final dataset of 2658 compounds was obtained. To create the best ML model for the virtual screening of these phytocompounds, we constructed different classification models using an ML approach. Six different classification models were developed. These included Discussion Stump, Random Forest, Random Tree, Rep Tree, J48 Tree, and PART rules. The models trained and validated by means of tenfold cross-validation on the training dataset to ensure reliability and generalisation. The performance of each model was evaluated using standard statistical metrics, as shown in the corresponding Supplementary Table S3. Among the models tested, the REP tree model showed the highest performance. It achieved the highest Kappa statistic value of 0.859, indicating a high level of agreement between the predicted classifications and the actual classes. A value of 1 in Kappa statistics represents perfect agreement, and the results from the REP tree model were close to this ideal. Additionally, this model also showed the lowest root mean square error value of 0.25, indicating fewer deviations between predicted and actual outcomes. The REP tree model also showed the highest overall accuracy, attaining 92.95%, surpassing all the other models. Furthermore, the sensitivity and specificity of the REP Tree model were found to be 97% and 88%, respectively, which were the highest amongst all the classifiers. In contrast, the Randomized Tree classifier model showed the lowest sensitivity and specificity values at 66% and 68%, respectively.

Further, to access the model, the Receiver Operating Characteristics (ROC) curve used for the evaluation method helps to visualise the performance of binary classification models at different discrimination thresholds. The ROC curve for the REP Tree model showed the strongest relationship between the true positive rate (sensitivity) and the false positive rate, thus indicating its superior classification ability. It recorded the highest ROC value among all models, while the Random Tree model exhibited the lowest ROC performance. Based on the overall performance in all evaluated parameters, such as accuracy, sensitivity, specificity, RMSE, Kappa statistics and ROC curve, the REP Tree model was selected for virtual screening of the phytocompounds as illustrated in Supplementary Fig. S3. Using this model, it was observed that out of the 2658 screened natural compounds, 745 were classified as active, while 1913 were identified as inactive. All 745 active compounds were selected for subsequent screening and further analysis.

### Pharmacokinetic and pharmacodynamic profiling

After initial virtual screening, a total of 745 compounds were screened for ADMET profiles to narrow them down based on the key pharmacokinetic and toxicity parameters. The first step we evaluated the Lipinski Rule of five, checking molecular weight, logP, number of hydrogen bond acceptor and donors. Compounds violating more than one of these criteria are predicted to have poor absorption and permeability. Based on this, 744 compounds satisfied the Lipinski rule and were retained for further screening. Next, we evaluated Human Intestinal Absorption (HIA). Compounds with an absorption rate below 30% are considered poorly absorbed. Using a threshold of 0.4, a total of 324 compounds were found to meet the criteria. Following, we checked the interaction with P-glycoprotein (P-gp), a key membrane protein involved in drug transport. Compounds with P-gp substrate scores within a range of 0.7 were considered likely to avoid efflux, and 309 compounds satisfied this condition. We then assessed Plasma Protein Binding (PPB), as compounds with high protein binding (above 90%) may have limited therapeutic activity due to reduced free drug availability. Based on this, compounds with PPB values less than 90% were selected, reducing the total to 132 compounds. To ensure that the compounds could effectively reach the central nervous system, we screened for blood-brain barrier (BBB) permeability using a threshold of 0.7, identifying 126 compounds with potential BBB penetration, suggesting their potential to exhibit good treatment efficacy against bacterial meningitis. Subsequently, we analysed plasma clearance (CL_plasma), which is important for determining drug dosage and exposure. Compounds with a clearance rate around 0.5 were considered optimal, and their step filtered the number of compounds down to 67. Finally, we performed toxicity prediction focusing on compounds showing predicted Toxicity classes higher than 4 and having LD50 values above 2000 mg/kg. Compounds showing predicted toxicity in hepatotoxicity, carcinogenicity, respiratory and mutagenicity were considered. This step reduced the number of compounds to five that met all ADMET and toxicity criteria and were selected for further analysis (Supplementary Table S4).

### Density functional theory analysis

**Fig. 2:**
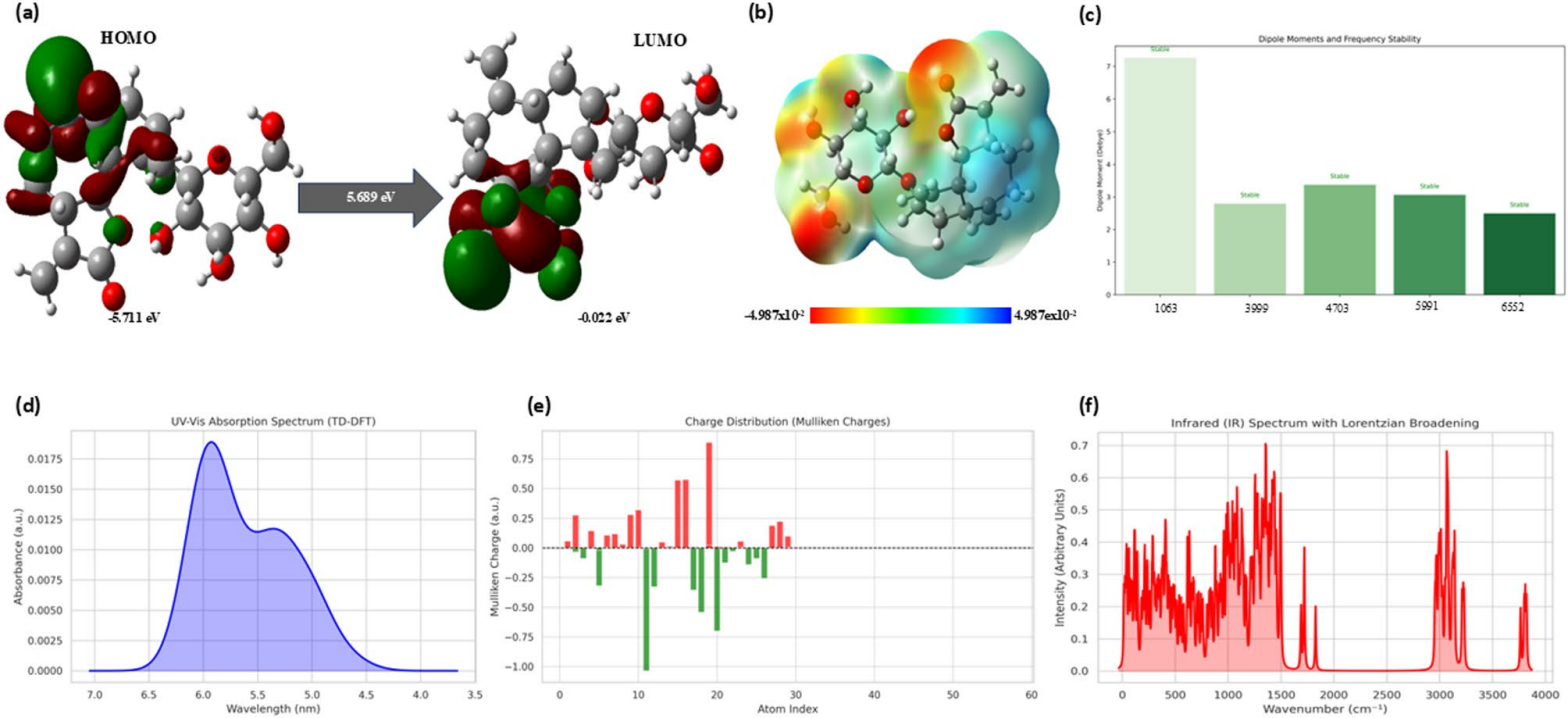
Density functional theory (DFT) and time-dependent DFT (TD-DFT) analysis of glucozaluzanin C (**a**) Frontier molecular orbitals (FMOs) depicting the HOMO (− 5.71 eV) and LUMO (− 0.022 eV) distributions with an energy gap of 5.689 eV, indicating electronic excitation potential (**b**) Molecular Electrostatic Potential (MEP) surface showing charge distribution across the molecular surface; red and blue regions represent electron-rich and electron-deficient areas, respectively. (**c**) Dipole moment and vibrational frequency analysis indicating molecular stability across normal modes (values shown for selected modes) (**d**) UV–Vis absorption spectrum simulated using TD-DFT, showing maximum absorbance in the range of 6.0–6.5 nm (**e**) Mulliken charge distribution analysis across atomic indices, highlighting regions of electron donation (negative charge) and acceptance (positive charge). (**f**) Simulated Infrared (IR) spectrum with Lorentzian broadening, identifying key vibrational modes contributing to molecular characterization.

The electronic and structural properties of the selected compounds are closely related to their frontier molecular orbitals (FMOs), specifically the HOMO and the LUMO. These orbitals play a crucial role in defining the electron-donating and electron-accepting capacities of ligands. Understanding the energy difference between HOMO and LUMO levels is an important approach for assessing the chemical reactivity, kinetic stability and overall molecular stability of the compounds. Normally, a smaller HOMO-LUMO energy gap is associated with higher chemical reactivity and increased potential for biological activity, whereas a larger energy gap is associated with higher molecular stability and reduced reactivity. The five compounds that were screened from the ADMET were used for the prediction of their molecular stability and to understand their kinetic stability and chemical reactivity based on their FOMO structures. The compounds evaluated were 1063; 3999; 4703; 5991 and 6552. The corresponding HOMO-LUMO energy gaps for these compounds were found to be 0.68878 eV, 4.5843 eV, 4.1978 eV, 4.6024 eV, 4.0354 eV, respectively. A lower energy gap is associated with strong chemical reactivity and potential biological activity, whereas a larger gap suggests a more stable ad less reactive molecule. Based on the HOMO and LUMO energies, additional chemical parameters including Ionization Potential (IP), Electron Affinity (EA), Electronegativity (x), chemical hardness (ƞ), Chemical Softness (S) were calculated and are summarised in the Table 1. The HOMO and LUMO molecular orbital distributions, along with the molecular electrostatic potential (MEP) maps of the compound, is depicted in the Fig. 2a and b. In the MEP maps, blue colour indicates regions of significant positive electrostatic potential (electron-deficient areas), whereas the red colour indicates electron-rich regions. Electronegative atoms, such as oxygen and carbon, were observed in the red regions. Nucleophilic species preferentially target regions with higher negative electrostatic potential (red zones), while electrophilic species are attracted to areas with lower positive potential (blue zones). It was observed that the compound 1063 showed higher electronegativity and electrophilicity values compared to the other compounds, further supporting its potential for strong interactions and reactivity. The MEP analysis provided insights into the nucleophilic and electrophilic regions, with nucleophilic reactions associated with blue zones and electrophilic reactions linked to red zones. These observations were further supported by Time-Dependent Density Functional Theory (TD-DFT) based Ultraviolet-Visible (UV-Vis) absorption analysis, which indicated significant electronic transitions, Mulliken charge distribution highlighting polarised reactive centres, Infrared (IR) vibrational spectra confirming no imaginary frequencies and vibrational stability and the high dipole moment suggesting strong polarity and structural stability (Fig. 2c and f, Supplementary Figs. S4–S7).

**Table 1:** DFT- calculated electronic parameters of phytocompounds for PBP2x screening.

Parameters (eV)	IMPHY001063	IMPHY003999	IMPHY004703	IMPHY005991	IMPHY006552
HOMO	− 5.71087	− 6.2074	− 6.0967	− 5.7147	− 5.8339
LUMO	− 0.022092	− 1.6231	− 1.8989	− 1.1123	− 1.7985
Energy Gap (ΔE	5.689	4.584	4.198	4.602	4.035
Ionization potential (IP)	5.711	6.207	6.097	5.715	5.834
Electron affinity (EA)	0.022	1.623	1.899	1.112	1.799
Chemical potential (µ)	− 2.86648	− 3.91525	− 3.9978	− 3.4135	− 3.8162
Global hardness (η)	2.844	2.292	2.099	2.301	2.018
Softness (S) (eV^−1^)	0.352	0.436	0.476	0.434	0.496
Electronegativity (χ)	2.866	3.915	3.998	3.414	3.816
Electrophilicity index (ω)	1.445	3.343	3.805	2.531	3.610

### Molecular docking and intermolecular interactions analysis

Molecular docking analysis was conducted to evaluate the binding potential of selected phytocompounds with the active site residues of the PBP2x protein. The active binding sites of PBP2x included two conserved motifs: STMK and KSG. The STMK site comprised the residues SER337, THR338, MET339, LYS340, whereas the KSG site included LYS547, SER548, and GLY549. These residues were selected based on their structural proximity to the ligand-binding pocket and their functional relevance to the catalytic activity of PBP2x. To assess the effect of mutations at these active sites, five mutants of PBP2x were used in docking analysis along with five compounds. Amoxicillin was used as the reference compound to compare the binding energies and interaction profiles. In the wild-type PBP2x, Amoxicillin exhibited a binding energy of − 7.73 kcal/mol. For the STMK motif mutations, T338A, T338G, and T338P, the binding energies of Amoxicillin were − 7.79 kcal/mol, − 6.86 kcal/mol, and − 7.76 kcal/mol, respectively. In case of KSG mutations K547G and K547T, the binding energies were observed to be − 7.77 kcal/mol, − 7.28 kcal/mol, respectively, when docked with Amoxicillin. Hydrogen bond analysis revealed that in the wild-type PBP2x-Amoxicillin, four hydrogen bonds were formed with the residues SER337, THR526, LYS340, and SER395. In the K547G mutant, Amoxicillin formed five hydrogen bonds involving THR550, GLY567, SER548, SER395, and ASN397, although one unfavourable bond was also observed. Similarly, in the K547T variant, Amoxicillin formed two hydrogen bonds with GLY597 and THR550. For STMK site mutations, Amoxicillin formed two hydrogen bonds with SER337 and TRP374 in the T338A mutant, including an unfavourable interaction. In the T338G mutant, four hydrogen bonds were observed with THR550, SER337, GLU378 and TRP374. The T338P mutation resulted in three hydrogen bonds with SER337, THR526 and TRP374, along with one unfavourable bond.

All five phytocompounds were then docked with the five mutated forms of PBP2x. The docking scores range from − 8.22 to − 2.28 kcal/mol; the values are given provided within the Supplementary Table S5. Among these, Glucozaluzanin C (Compound ID: 1063) demonstrated the lowest binding energy of − 8.22 kcal/mol, indicating the strongest interaction with PBP2x. The docking results of Glucozaluzanin C revealed a total of five hydrogen bonds in the wild-type complex. Specifically, in the T338A mutant, Glucozaluzanin C formed four hydrogen bonds, primarily with SER337 and THR550. In the T338G mutant, it formed seven hydrogen bonds involving SER337, THR550, and GLU978. For the T338P mutant, Glucozaluzanin C formed six hydrogen bonds with SER395, LYS340, THR550 and SER337. In the K547G mutant, Glucozaluzanin C formed four hydrogen bonds that are THR526, GLY549, THR550, and SER571. Similarly, the K547T mutant formed 2 hydrogen bonds with THR526 and SER571. The comparative interaction profiles of Amoxicillin and Glucozaluzanin C with wild-type and mutated PBP2x proteins are presented in Supplementary Figs. S8 and S9, while the corresponding docking scores and interacting residues are summarised in Supplementary Table S6. The docking analysis demonstrated that Glucozaluzanin C shared several key binding residues with Amoxicillin, especially in the mutated binding pockets, suggesting its potential to retain binding affinity even in the presence of active site mutations. Glucozaluzanin C was found to effectively bind with the active site of mutated PBP2x and showed favourable binding energies and interactions. Based on the docking scores, interaction patterns and DFT analysis, Glucozaluzanin C was selected for further evaluation through molecular dynamic simulations.

### Dynamics simulations analysis

MD simulations were performed for 100 ns to evaluate the molecular behaviour and dynamics of the top docked compound, Glucozaluzanin C, in complex with both the wild-type and mutants of PBP2x. The resulting trajectories were analysed using multiple MD parameters. The analysis provided a comprehensive estimation of the structural stability and conformational fluctuations of the complexes. The RMSD values, which indicate the structural deviation of the complex over time, showed that all systems stabilised, with RMSD values ranging from 0.21 to 0.26 nm, indicating high structural stability. RMSD values less than 0.3 nm are considered acceptable for stable simulations. The average RMSD for each complex are provided in the table, and motif-wise fluctuations. MD simulations were also performed for the apo forms of the wild-type and mutants PBP2x proteins to evaluate baseline structure dynamics. The simulation results, including RMSD, RMSF, hydrogen bond ad SASA are provided in the Supplementary Fig. S10 for comparative reference. Further, RMSF analysis over the 100 ns simulation provided insights into residue-level flexibility. The wild type showed moderate fluctuations, particularly in loop regions around residue numbers ~ 300 and ~ 500. Mutants generally displayed reduced fluctuations at catalytic motifs, indicating localised rigidity that could favour ligand binding. RMSF values for PBP2x in complex with Glucozaluzanin C ranged from 0.11 to 0.14 nm. The RMSF profile between residues ~ 92–189 corresponds to an unresolved segment in the crystal structure. As confirmed by sequence alignment (Supplementary Fig. S11), this region lies outside the pencilling binding transpeptidase domain (residues 289–609) and does not influence the functional dynamics of the protein. Prior studies have shown that simulations of partially resolved protein remain valid when missing loops are distant from the active domain^65,66^. Hydrogen bond analysis showed that Glucozaluzanin C formed up to six hydrogen bonds with the wild type, several of which remained stable. In the K547G mutant, five hydrogen bonds were observed, with three consistent throughout the simulation. In K547T, six bonds formed, with one bond stable throughout the simulation, and more bonds forming towards the end. In the T338A mutant, the complex formed seven hydrogen bonds, with two remaining stable. T338G showed six hydrogen bonds with three stable ones, and T338P formed seven hydrogen bonds, with three being constant.

**Fig. 3:**
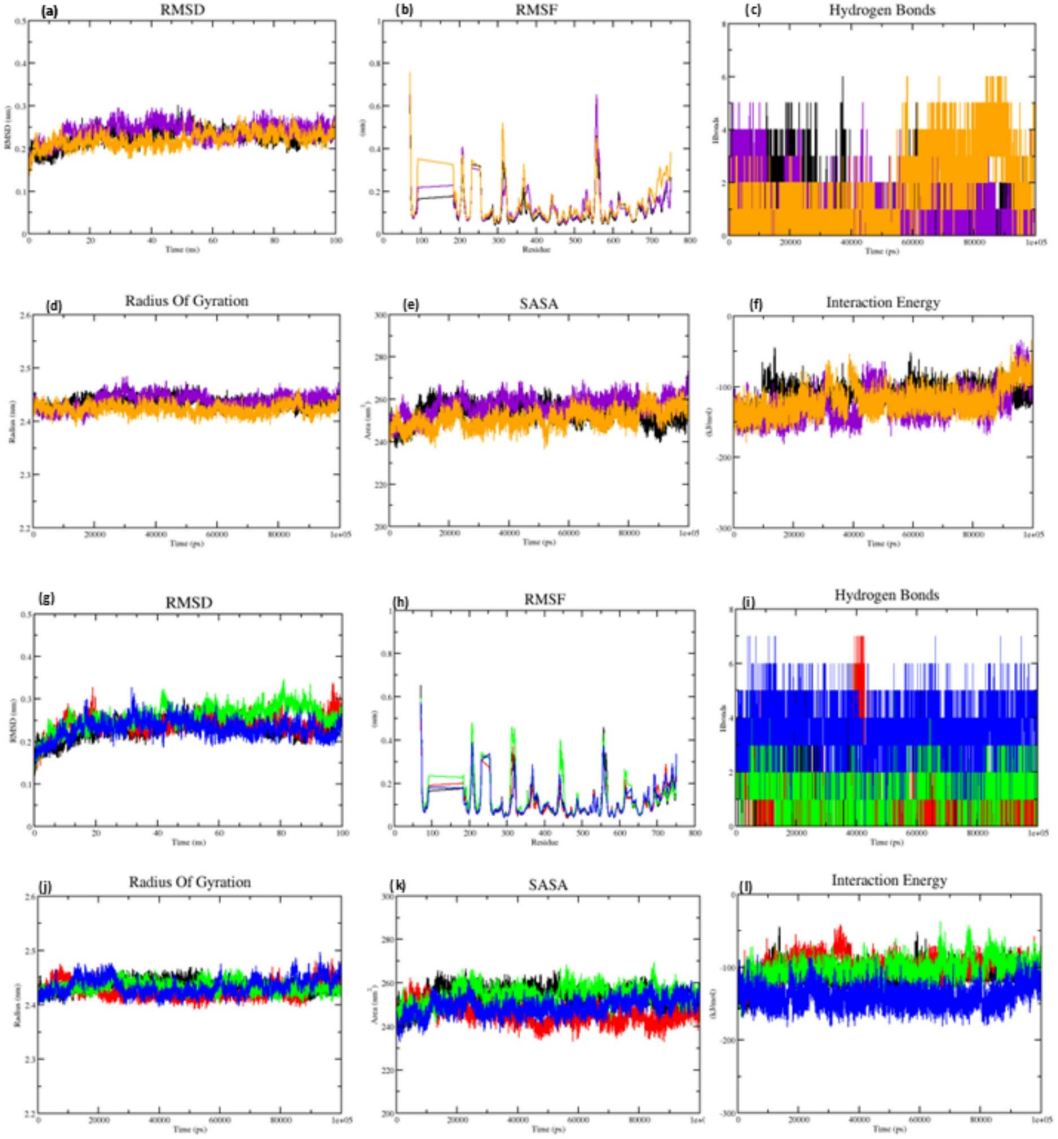
Molecular dynamics simulations for KSG motif (wild-type complex (black), mutant K547G complex (violet) and mutant K547T complex (orange)) and STMK motif (wild-type complex (black), mutant T338A complex (red), mutant T338G complex (green), and mutant T338P complex (blue)) (**a**) RMSD of SMTK motif (**b**) RMSF of SMTK motif (**c**) Number Hydrogen bond of SMTK motif (**d**) Radius of Gyration of SMTK motif (**e**) SASA of SMTK motif (**f**) Interaction Energy of SMTK motif (**g**) RMSD of KSG motif (**h**) RMSF of KSG motif (**i**) Number Hydrogen bond of KSG motif (**j**) Radius of Gyration of KSG motif (**k**) SASA of KSG motif (**l**) Interaction Energy of KSG motif of KSG motif (**c**) SASA of KSG motif (**e**) Interaction Energy of KSG motif.

The radius of gyration (RoG) was used to evaluate structural efficiency during the simulations, where the lower RoG values specify more stable and compact protein structures. Glucozaluzanin C-PBP2x complexes showed minimal fluctuation in RoG values, ranging from 2.42 to 2.44 nm across all systems, SASA analysis also revealed low variation in the solvent-accessible surface area, suggesting that the presence of Glucozaluzanin C helped maintain protein folding and surface stability throughout the 100ns simulation. Interaction energy calculated using short-range Lennard–Jones interactions (LJIE) further supported strong ligand binding. The wild-type complex showed an interaction energy of − 112.14 KJ/mol with a 2.4 error rate. For mutants K547G and K547T, interaction energies were − 127.29 KJ/mol and − 120.37 KJ/mol, with error rates of 6.70 and 5.60, respectively. T338A, T338G, and T338P showed interaction energies of − 101.34, − 105.76 and − 141.68 KJ/mol, with corresponding errors of 3.50, 2.70 and 1.70 and the results are depicted in Fig. 3a and l. These values indicate effective binding in all systems (Table 2). PCA was conducted to study overall structural motion and conformational dynamics (Supplementary Fig. S12). FEL based on the first two principal components (PC1 and PC2) were generated. The wild-type complex revealed a deep energy minimum, indicating conformational stability. Mutants K547G and K547T showed low energy basins with retained structural stability. The mutants T338A, T338G, and T338P showed well-formed energy wells, supporting functionally relevant conformational changes while maintaining favourable interactions with Glucozaluzanin C. These results indicate that the PBP2x-Glucozaluzanin C complexes exhibit consistent, stable dynamics and binding across wild-type and mutant systems, particularly within the transpeptidase domain (Supplementary Fig. S13).

**Table 2:** Molecular dynamics simulation parameters and MM/GBSA energy profile of Glucozaluzanin C complexes with wild-type and mutant PBP2x.

Molecular dynamics simulation parameters								MM/GBSA energy profile						
Complexes	Avg. RMSD (nm)	Avg. RMSF (nm)	Avg. ROG (nm)	Avg. ASA (nm^2^)	Avg. IE (kJ/mol)	Avg. Rate	H-Bond	ΔVD -WAALS	ΔEEL	ΔEGB	ΔESURF	ΔGGAS	ΔGSOLV	ΔTotal
WT-1063	0.23	0.11	2.44	253.49	− 112.14	2.40	6	− 23.64	− 15.94	31.31	− 3.56	− 39.58	27.74	− 11.84
MT-K547G-1063	0.24	0.12	2.44	256.85	− 127.29	6.70	5	− 27.93	− 25.04	41.97	− 4.21	− 52.97	37.76	− 15.2
MT-K547T-1063	0.22	0.14	2.42	250.89	− 120.37	5.60	6	− 28.95	− 22.27	40.32	− 4.56	− 51.23	35.76	− 15.46
MT-T338A-1063	0.21	0.11	2.43	245.54	− 101.34	3.50	7	− 28.37	− 6.22	23.01	− 3.91	− 34.59	19.1	− 15.49
MT-T338G-1063	0.26	0.13	2.43	254.06	− 105.76	2.70	6	− 34.36	− 14.12	32.97	− 5	− 48.48	27.96	− 20.51
MT-T338P-1063	0.23	0.11	2.43	248.93	− 141.68	1.70	7	− 32.55	− 20.9	37.98	− 5	− 53.45	32.98	− 20.48

### MM-GBSA analysis

The binding free energy (∆G) for the best protein-ligand complexes was evaluated using the gmx MMPBSA. Additionally, the gmx MMPBSA ana utility was used to perform component analysis and generate visual representations in the form of graphs. These graphs depict the overall binding energy components, including gas-phase energy (∆G gas), solvation energy (∆G solvation) and total binding free energy (∆G total), as well as specific individuals contributing such as van der Waals (∆G EvdW) and electrostatic (∆G Eelec) interactions. (Supplementary Fig. S14) represents comparative values for all six protein-ligand complexes evaluated. The wild-type PBP2x—Glucozaluzanin C complex exhibited a ∆G total of − 11.84 kcal/mol. Among the three mutants in the STMK motif (T338A, T338G, and T338P), the calculated ∆G total values were − 15.20 kcal/mol, − 15.46 kcal/mol and − 15. 49 kcal/mol respectively. For the KSG mutants (K547G and K547T), stronger binding affinities were observed with ∆G total values of − 20.51 kcal/mol and − 20.48 kcal/mol, respectively (Table 2) These results suggest that Glucozaluzanin C forms favourable and stable complexes with both wild-type and mutant forms of PBP2x. The improved binding affinities observed in the mutant complexes can be assigned to enhance van der Waals interactions, favourable gas-phase contributions, and supported electrostatic interactions. These findings confirm that Glucozaluzanin C exhibits acceptable ∆G values and forms stable complexes with the mutants of PBP2x. To further understand the residue-level contributions to binding, per-residue MM/GBSA energy decomposition analysis was performed. Notably, residues such as SER395, LYS496 and THR550 contributed significantly to the overall binding energy across all complexes. The residues appear to be key stabilizing elements within the binding pockets (Supplementary Fig. S15).

## Discussion

PBP2x plays a crucial role in the development of the β-lactam resistance in *S. pneumoniae*, primarily due to its essential function in catalysing the transpeptidation reaction during bacterial cell wall biosynthesis. Mutations within the conserved motifs of PBP2x weaken its ability to bind β-lactam antibiotics, thereby reducing susceptibility and contributing to resistance. Specifically, these alterations can also affect the proteins’ enzymatic function, potentially leading to defects in the cell wall synthesis^10^. Among the widely investigated mutations, at position T338, located adjacent to the active site S337 in the STMK catalytic motif, is frequently substituted with T338A/G/P. These mutations have been experimentally validated in vitro and are known to influence β-lactam binding^67^. Similarly, mutations in the KSG catalytic motif, such as the substitution of K547 with K547G/T, occur at the end of the β3 loop and are associated with increased acylation efficiency towards β-lactam^7,12^. These changes, being situated in conserved active sites, can substantially alter the protein’s structural and functional integrity. Recent regional studies reported that PBP2x mutations, particularly T338 and K547 substitutions, are strongly associated with respiratory and invasive infections such as pneumonia, meningitis and septicaemia. Serotypes 19A, 6b and 23F, which carry these mutations, are the most frequently detected in clinical cases across different countries such as China, India, Japan, South Korea, Vietnam, and parts of Europe, where antimicrobial resistance is a growing public health challenge. The widespread presence of these resistant variants highlights the critical need for new inhibitors targeting the structurally altered PBP2x^68^.

In this study, we specifically analysed mutations within the STMK and KSG motifs, particularly T338A/G/P, K547G/T. All selected mutations have been reported in the literature as clinically relevant and tested. Computational evaluation indicated that each of these mutations had a deleterious effect on PBP2x functionality. These findings highlight the urgent need for novel therapeutic agents targeting β-lactam resistant *S. pneumoniae* strains. The increasing prevalence of multidrug-resistant organisms is largely driven by the widespread use of antibiotics, which accelerates the selection of resistance factors^69^. Natural compounds, especially those derived from plants, offer a promising alternative for antimicrobial drug discovery due to their broad-spectrum biological activities. These include antibacterial, antioxidant, anti-inflammatory, anticancer, immunomodulatory, and neuroprotective effects^70^. Several phytochemicals have demonstrated efficacy against *S. pneumoniae*, particularly in conjunction with or as alternatives to β-lactam antibiotics^71,72^. Phenolic compounds are of particular interest because of their ability to bind to bacterial virulence factors such as PBPs, inhibit biofilm formation, and disrupt bacterial membranes. These effects have been documented in Gram-positive and Gram-negative bacteria^73,74^. In this study, we employed ML models to screen a library of plant-based compounds against mutant variants of PBP2x. The application of ML improved in the identification of phytochemicals with potential inhibitory activity against all five resistance-associated PBP2x mutations.

Previous studies have demonstrated the effectiveness of ML in predicting active compounds against protein targets associated with drug resistance^20,75^. The use of ROC curves and the AUC-ROC provided robust metrics for model performance evaluation, distinguishing active from inactive compounds^76^. Genomic surveillance tools, as shown by Bilal and team, show how unsupervised ML algorithms can be used to study evolutionary patterns and predict susceptibility based on PBP sequence variation^77^. In our study, ML algorithms were employed to filter active compounds based on their predicted activity profiles, further evaluated through ADMET profiling DFT analysis. Among the top compounds, Glucozaluzanin C, a guaiane-type sesquiterpene lactone isolated from *Elephantopus scaber*, showed the most favourable binding energy and was selected for further molecular docking and simulation studies. DFT analysis for all five lead compounds revealed low HOMO-LUMO energy gaps, indicative of high chemical reactivity and potential biological activity. In particular, the DFT-derived electronic properties of the ligands offer detailed insights into their biological activity and binding behaviour. Compound Glucozaluzanin C, which exhibited a high HOMO-LUMO energy gap (5.69 eV), suggests high polarizability and stability. It also demonstrated strong binding in docking (-8.22 kcal/mol) and favourable MM/GBSA binding free energy (-11.84 kcal/mol). The high dipole moment (7.3D) further specifies enhanced complementary electrostatic interaction with charged residues in the binding pockets. ESP analysis showed electron-rich zone near hydroxyl and carbonyl groups, aligning well with the hydrogen-bond donor/acceptor regions in PBP2x. Per-residue MM/GBSA decomposition analysis confirmed consistent contribution to binding stabilization. Thes results support a detailed understanding of how electronic descriptors explain PBP2x – Glucozaluzanin C complex interaction. MD simulations further confirmed the structural stability of PBP2x – Glucozaluzanin C complex, as indicated by the consistent RMSD, RMSF, RoG and SASA over the period^78,79^. To better understand the global motion and dynamic behaviour of the protein-ligand complex, PCA was performed on the trajectory. The first few eigenvectors accounted for the majority of the collective motions, confirming that Glucozaluzanin C binding results in a stable and restricted conformational landscape compared to the unbound state. Furthermore, MM/GBSA free energy calculations were employed to estimate the binding affinity, indicating highly favourable interactions, particularly in T338P and K547G variants, suggesting strong binding affinity ad stability of the complex. Glucozaluzanin C has previously exhibited antimicrobial effects against *Staphylococcus aureus*, *Pseudomonas aeruginosa*, *Escherichia coli*, *Bacillus subtilis*, and *Aspergillus niger*^80–83^. Although direct evidence of its efficacy against *S. pneumoniae* remains limited, its traditional use and broad-spectrum antimicrobial properties suggest the need for further investigation^84^. ADMET evaluation confirmed Glucozaluzanin C’s favourable pharmacokinetic profile, including good absorption, non-toxic behaviour and compatibility as a P-glycoprotein substrate. While these computational findings are encouraging, experimental validation is still needed. Glucozaluzanin C has not yet been tested in vitro and in vivo against *S. pneumoniae*, and its inhibitory activity remains to be confirmed through biological studies. Future work will focus on validating the efficacy of the compound using in vivo experimental models.

Overall, this study demonstrated that Glucozaluzanin C exhibits strong potential as an inhibitor of β-Lactam resistant PBP2x in *S. pneumoniae.* The integration of ML-based screening, DFT electronic characterisation, MM/GBSA energetic evaluation, PCA-based motion analysis, and MD simulation presents a comprehensive and effective strategy for natural product-based drug discovery. Compared to traditional QSAR approaches, ML models offer greater accuracy and efficiency with smaller datasets^85,86^. This emphasizes the values of ML-driven pipelines for early-stage identification of phytochemical leads in the context of antibiotic resistance. DFT analysis provided additional insights into the electronic properties and reactivity of Glucozaluzanin C, with moderate HOMO-LUMO energy gaps indicating favourable electron transfer characteristics and potential biological activity^87^. The absence of imaginary vibrational frequencies confirmed that the optimised geometry represents a true energy minimum, supporting the structural stability of the compound^88^. Electrostatic potential surface mapping further revealed key regions of nucleophilic and electrophilic potential, which are relevant to interactions with the PBP2x active site^78^. Geometry optimisation ensured a reliable molecular conformation for downstream analysis. Finally, molecular docking simulations targeting key PBP2x resistance mutations (T338A, T338G, T338P, K547G, K547T) supported that Glucozaluzanin C forms stable hydrogen bonds as a competitive inhibitor^89,90^.

## Conclusion

The findings of this study revealed the important mutations (T338A/G/P and K547G/T) in the PBP2x protein of *S. pneumoniae*, which are known to reduce the effectiveness of β-lactam antibiotics. Using a combination of machine learning, DFT analysis, molecular docking, and dynamic simulations, we screened and evaluated plant-based compounds for their ability to bind to the resistant protein PBP2x. Among the compounds tested, Glucozaluzanin C showed the most promising results. It was able to bind effectively to all five PBP2x mutants, showed stable behaviours during simulations, and had favourable electronic and pharmacokinetic properties. Its known antimicrobial activity and strong interaction with PBP2x mutants make it a good candidate for further study. The computational approach used in this study provides a useful method for identifying natural compounds that could help in the antibiotic-resistant organisms. These results may support the future development of Glucozaluzanin C and related molecules as therapeutic agents against β-lactam resistant *S. pneumoniae.*

## Supplementary Information

Below is the link to the electronic supplementary material.

Supplementary Material 1

Supplementary Material 2

## Data Availability

The protein structure analysed for this study was retrieved from the Protein Data Bank (PDB) https://www.rcsb.org/, and the phytocompounds were obtained from the IMPPAT Database https://cb.imsc.res.in/imppat/.
