# Supplementary material for: Machine learning-based virtual screening and density functional theory characterisation of natural inhibitors targeting mutant PBP2x in Streptococcus pneumoniae
Source: Sci Rep. 2025 Nov 7;15:39164. doi: 10.1038/s41598-025-24222-1 (PMC12595054; doi:10.1038/s41598-025-24222-1)
Supplement: Supplementary file 1 — Supplementary Material 1 [file 41598_2025_24222_MOESM1_ESM.docx]

**Supplementary Table S1:** Predicted deleterious effects of T338 and K547 mutations using pathogenicity predictors

| **Mutation** | **PhD-SNP** | **SIFT** | **SNAP** | **Meta-SNP** | **Provean** |
| --- | --- | --- | --- | --- | --- |
| T338A | Deleterious | Deleterious | Deleterious | Deleterious | Deleterious |
|  | 0.574 | 0.04 | 0.725 | 0.617 | -3.937 |
| T338G | Deleterious | Deleterious | Deleterious | Deleterious | Deleterious |
|  | 0.774 | 0 | 0.735 | 0.744 | -6.114 |
| T338P | Deleterious | Deleterious | Deleterious | Deleterious | Deleterious |
|  | 0.846 | 0 | 0.745 | 0.729 | -5.155 |
| K547G | Deleterious | Deleterious | Deleterious | Deleterious | Deleterious |
|  | 0.735 | 0 | 0.835 | 0.772 | -6.961 |
| K547T | Deleterious | Deleterious | Deleterious | Deleterious | Deleterious |
|  | 0.817 | 0 | 0.835 | 0.794 | -5.967 |

**Supplementary Table S2**: Predicted destabilizing changes and Molecular Interaction Alterations Induced by PBP2x Mutations

| **IDs** | **Predicted ∆∆G** | **Interactions lost** | | | | **Interaction formed** | | | |
| --- | --- | --- | --- | --- | --- | --- | --- | --- | --- |
|  |  | **Hydrogen Bond** | **Hydrophobic** | **Carbonyl** | **Polar** | **Clash** | **VDW** | **Hydrophobic** | **Polar** |
| **K547G** | -2.29 | SER395, SER337 | VAL341, PHE392, MET515, VAL573 | ALA572 | SER395 | NONE | NONE | NONE | NONE |
| **K547T** | -1.98 | THR338, SER395, SER337 | VAL573 | NONE | SER395 | NONE | NONE | VAL518 | NONE |
| **T338A** | -1.83 | LYS547, PRO335, MET461 | LYS573, TYR586 | NONE | PRO335, SER571 | LYS340 | NONE | MET461, PHE465 | NONE |
| **T338G** | -2.55 | PRO335, MET461, LYS547 | VAL573, TYR586 | NONE | SER571, MET461, TYR586, PRO335 | NONE | NONE | NONE | LYS340 |
| **T338P** | -0.23 | GLY336, SER547, MET461 | TYR586, VAL573 | NONE | MET461, SER571, GLY336 | NONE | GLY336, GLY549, SER571, VAL341 | MET461, PHE465 | NONE |

**Supplementary Table S3**: Model performance metrics for machine learning-based compound screening

| **Models** | **Decision stump** | **Random Forest** | **Random Tree** | **Rep Tree** | **J48** | **PART** |
| --- | --- | --- | --- | --- | --- | --- |
| **Correctly classified instances/ACC (%)** | 87.3239 | 83.0986 | 67.6056 | 92.9577 | 85.9155 | 85.9155 |
| **Kappa statistics** | 0.7459 | 0.6622 | 0.3522 | 0.859 | 0.718 | 0.718 |
| **Mean absolute error (MAE)** | 0.1764 | 0.2755 | 0.3239 | 0.0864 | 0.1445 | 0.1417 |
| **Root Mean Squared Error (RMSE)** | 0.3373 | 0.3464 | 0.5692 | 0.25 | 0.3728 | 0.3698 |
| **MCC** | 0.753 | 0.663 | 0.352 | 0.862 | 0.719 | 0.719 |
| **ROC** | 0.827 | 0.91 | 0.676 | 0.941 | 0.882 | 0.907 |
| **Recall** | 0.872 | 0.831 | 0.676 | 0.93 | 0.859 | 0.86 |
| **Error Rate/ incorrectly classified instances (%)** | 12.6761 | 16.9014 | 32.3944 | 7.0423 | 14.0845 | 14.0845 |
| **Precision** | 0.881 | 0.832 | 0.676 | 0.933 | 0.86 | 0.86 |
| **F-Measure** | 0.872 | 0.831 | 0.676 | 0.924 | 0.859 | 0.859 |
| **PRC Area** | 0.818 | 0.898 | 0.619 | 0.929 | 0.842 | 0.874 |
| **True Positives for Active** | 34 | 29 | 24 | 35 | 32 | 32 |
| **True Positives for Inactive** | 28 | 30 | 24 | 31 | 29 | 29 |
| **False Negative for Active** | 2 | 7 | 12 | 1 | 4 | 4 |
| **False Positives for Active** | 7 | 5 | 11 | 4 | 6 | 6 |

**Supplementary Table S4**: ADMET analysis of selected compounds targeting PBP2x mutants

| **Properties** | **IMPHY001063** | **IMPHY005991** | **IMPHY003999** | **IMPHY006552** | **IMPHY004703** |
| --- | --- | --- | --- | --- | --- |
| **Compound Name** | Glucozaluzanin C | 3,5-Dihydroxybenzoic acid | d-Tartaric acid | 2',4'-Dihydroxyacetophenone | D-Erythronolactone |
| **Lipinski Violations** | 0 | 0 | 0 | 0 | 0 |
| **pgp_substrate** | 0.296 | 0.029 | 0.053 | 0.261 | 0.427 |
| **HIA** | 0.000 | 0.002 | 0.003 | 0.037 | 0.000 |
| **BBB** | 0.010 | 0.007 | 0.187 | 0.218 | 0.001 |
| **PPB** | 69.742 | 37.822 | 14.829 | 88.095 | 16.852 |
| **cl-plasma** | 2.011 | 4.750 | 2.049 | 2.902 | 1.985 |
| **Predicted LD50 (mg/kg)** | 2000 | 2000 | 2497 | 2830 | 10600 |
| **Predicted Toxicity Class** | 4 | 4 | 5 | 5 | 6 |
| **Hepatotoxicity** | Inactive | Inactive | Inactive | Inactive | Inactive |
| **Respiratory** | Inactive | Inactive | Inactive | Inactive | Inactive |
| **Carcinogenicity** | Inactive | Inactive | Inactive | Inactive | Inactive |
| **Mutagenicity** | Inactive | Inactive | Inactive | Inactive | Inactive |

**Supplementary Table S5:** Docking analysis of Binding energy and inhibition constant for wild-type and mutant PBP2x complexes

| **ID** | | **WT_1QME** | **MT_K547G** | **MT_K574T** | **MT_T338A** | **MT_T338G** | **MT_T338P** |
| --- | --- | --- | --- | --- | --- | --- | --- |
| **AMO** | **Binding Energy** | -7.73 | -7.77 | -7.28 | -7.79 | -6.86 | -7.76 |
|  | **Inhibition Constant** | 2.16 μM | 2.02 μM | 4.63 μM | 1.96 μM | 9.42 μM | 2.04 μM |
| **1063** | **Binding Energy** | -8.22 | -8.1 | -8.04 | -8.04 | -8.15 | -8.21 |
|  | **Inhibition Constant** | 944.78 nM | 1.16 μM | 1.28 μM | 1.27 μM | 1.06 μM | 964.71 nM |
| **4703** | **Binding Energy** | 4.5 | -4.52 | -4.54 | -4.49 | -4.51 | -4.54 |
|  | **Inhibition Constant** | 504.54 nM | 484.54 μM | 473 μM | 507.52 μM | 492.94 μM | 472.11 μM |
| **5991** | **Binding Energy** | -4.98 | -4.51 | -4.55 | -5.03 | -5.14 | -4.92 |
|  | **Inhibition Constant** | 224.1 nM | 496.59 μM | 461.37 μM | 205.33 μM | 171.23 μM | 247 μM |
| **6552** | **Binding Energy** | -5.04 | -5.26 | -5.21 | -5.34 | -5.2 | -5.05 |
|  | **Inhibition Constant** | 201.24 nM | 138.88 μM | 150.8 μM | 121.85 μM | 153.28 μM | 199.44 μM |
| **3999** | **Binding Energy** | -3.8 | -2.7 | -2.28 | -3.07 | -3.03 | -3.16 |
|  | **Inhibition Constant** | 5.15 mM | 10.51 mM | 21.19 mM | 5.29 mM | 5.98 mM | 4.82 mM |

**Supplementary Table S6:** Molecular Docking results of Glucozaluzanin C and Amoxicillin with wild-type and PBP2x mutants

| **ID** | **Glucozaluzanin C** | | | | | | **Amoxicillin** | | | | | |
| --- | --- | --- | --- | --- | --- | --- | --- | --- | --- | --- | --- | --- |
|  | **BE (kcal/mol)** | **IC (μM)** | **H Bond** | **Hydro-phobic Bond** | **Other Inter-actions** | **Unfavour-able Bonds** | **BE (kcal/mol)** | **IC (μM)** | **H Bond** | **Hydro-phobic Bond** | **Other Inter-actions** | **Unfavour-able Bonds** |
| **Wild-Type** | -8.22 | 0.94 | THR526, SER548, THR550, SER571 | SER337, GLU378, SER395, MET527, THR536, GLY549, PHE570, ALA572, TYR595, GLY597, LEU600 | TRP374 | NONE | -7.73 | 2.16 | SER337, LYS340, SER395, THR526 | TRP374, ASN377, MET527, SER548, GLY549, THR550, ALA551, PHE570 | NONE | ASN397 |
| **MT-K547G** | -8.1 | 1.16 | THR526, GLY549, THR550, SER571 | SER337, GLU378, SER395, MET527, THR536, SER548, PHE570, ALA572, TYR595, GLY597, LEU600 | TRP374 | NONE | -7.77 | 2.02 | SER395, ASN397, SER548, THR550, GLY597 | LYS340, ASN377, THR526, MET527, GLY549, SER596 | TRP374, PHE570, TYR595 | SER337 |
| **MT-K574T** | -8.04 | 1.28 | THR526, SER571 | SER337, GLU378, SER395, THR536, MET527, SER548, GLY549, THR550, PHE570, ALA572, TYR595, GLY597, LEU600 | TRP374 | NONE | -7.28 | 4.63 | THR550, GLY597 | SER337, HIS394, SER395, THR526, SER548, GLY549, PHE570, TYR595, SER596 | TRP374 | NONE |
| **MT-T338A** | -8.04 | 1.27 | SER337, THR550 | GLY336, LYS340, ARG372, ASN377, TRP374, SER395, ASN397, GLN452, THR526, LYS547, SER548, GLY549, ALA551, GLN552, PHE570 | PHE450, TYR568 | NONE | -7.79 | 1.96 | SER337, TRP374 | THR550, GLY549, GLU378, SER548, SER395, LYS340, ASP373, PHE450, GLN452, GLN552, ALA551, LYS547, ASN397 | TYR568 | THR526 |
| **MT-T338G** | -8.15 | 1.06 | SER337, GLY549, THR550 | GLY336, LYS340, SER395, ASN397, GLN452, THR526, LYS547, SER548, ALA551, GLN552, TYR568, PHE570, SER571 | TRP374, TYR595 | NONE | -6.86 | 9.42 | SER337, TRP374, GLU378, THR550 | LYS340, ASP373, SER395, ASN397, GLN452, THR526, LYS547, SER548, GLY549, ALA551, GLN552 | TYR568 | NONE |
| **MT-T338P** | -8.21 | 0.96 | SER337, LYS340, SER395, THR550 | GLY336, ARG372, TRP374, ASN377, ASN397, GLN452, LYS547, GLY549, ALA551, GLN552 | PHE450, TYR568 | NONE | -7.76 | 2.04 | SER337, TRP374, THR526 | LYS340, ASP373, SER395, ASN397, PHE450, GLN452, LYS547, SER548, GLY549, THR550, ALA551, GLN552 | TYR568 | GLU378 |
