# Supplementary material for: Machine learning-based virtual screening and density functional theory characterisation of natural inhibitors targeting mutant PBP2x in Streptococcus pneumoniae
Source: Sci Rep. 2025 Nov 7;15:39164. doi: 10.1038/s41598-025-24222-1 (PMC12595054; doi:10.1038/s41598-025-24222-1)
Supplement: Supplementary file 2 — Supplementary Material 2 [file 41598_2025_24222_MOESM2_ESM.docx]

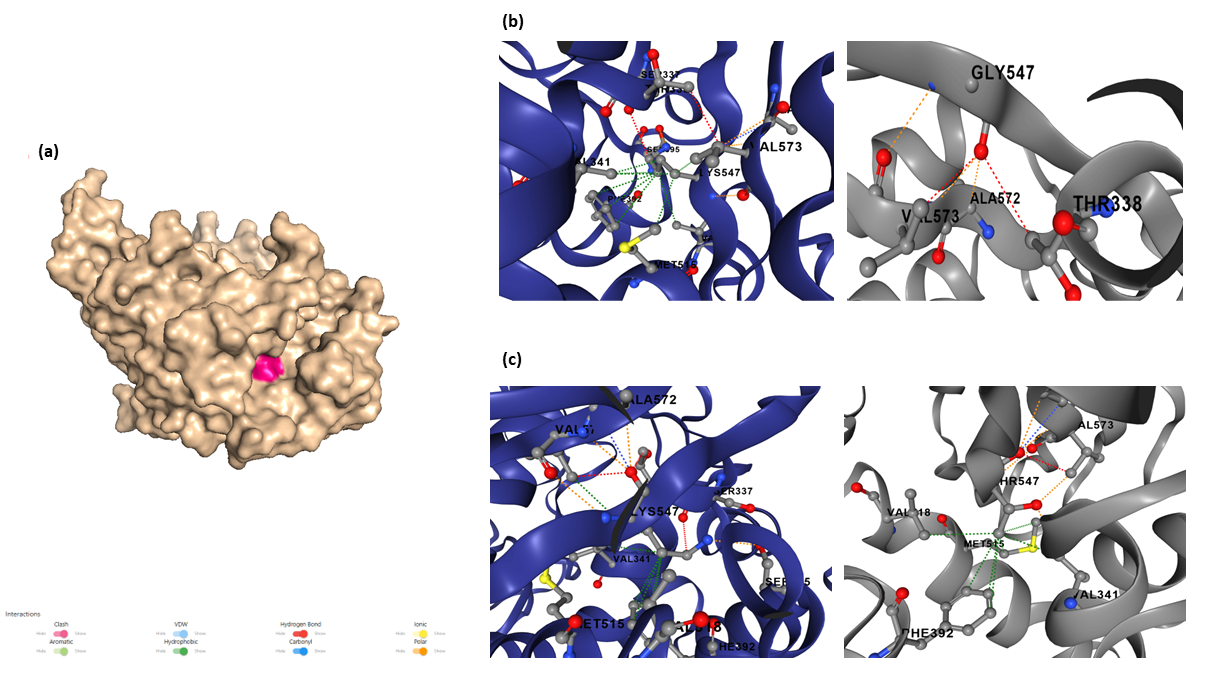
**Supplementary Figure S1:** Protein structure and stability **(a)** Surface representation of the protein structure showing active site KSG (magenta), buried within the binding pocket **(b)** Structural stability analysis of the K547G mutant were wildtype shown in blue, and the mutant show in gray **(c)** Structural stability analysis of the K547T mutant were wildtype shown in blue, and the mutant show in gray, indicating the changes in molecular interactions upon mutations


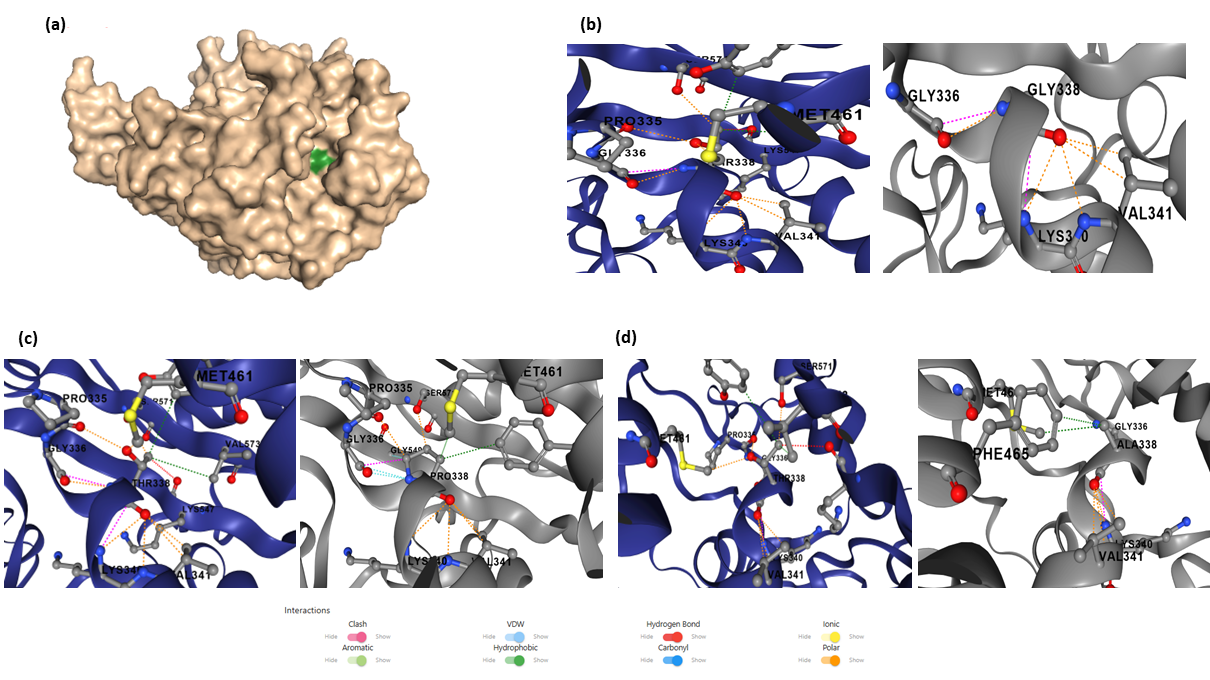


**Supplementary Figure S2:** Protein structure and stability **(a)** Surface representation of the protein structure showing active site STMK (green), buried within the binding pocket **(b)** Structural stability analysis of the T338G mutant were wildtype (blue), and the mutant (gray), **(c)** Structural stability analysis of the T338P mutant were wildtype (blue), and the mutant (gray) **(d)** Structural stability analysis of the T338A mutant were wildtype (blue), and the mutant (gray) indicating the changes in molecular interactions upon mutations


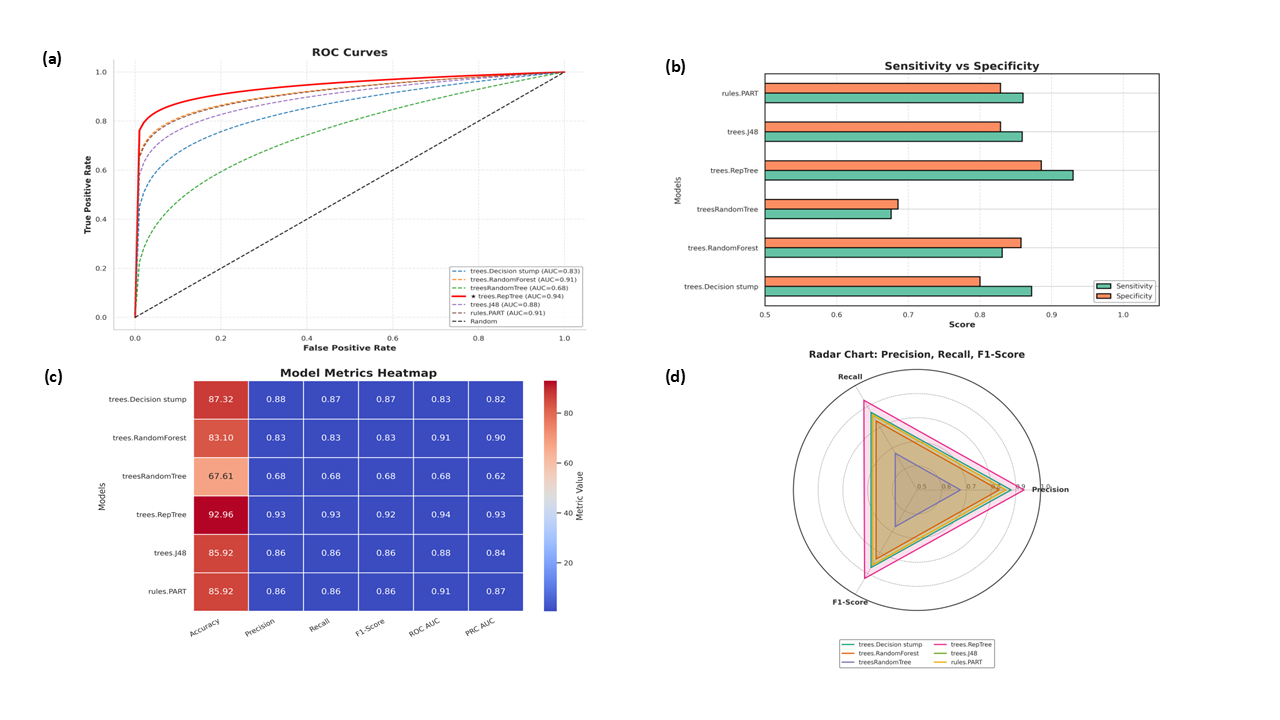
 **Supplementary Figure S3:** Performance comparison of various machine learning models using multiple evaluation metrics. **(a)** ROC curves showed the classification performance of each model, with REPTree achieving the highest AUC. **(b)** Bar lot represents the sensitivity and specificity, indicating the overall balance of each model. **(c)** Hetmap summarizes key metrics across all parameters, confirming REPTree as the most effective model. **(d)** The radar chart visualizes precision, recall, and F1-score, highlighting the consistent performance of the models across metrics


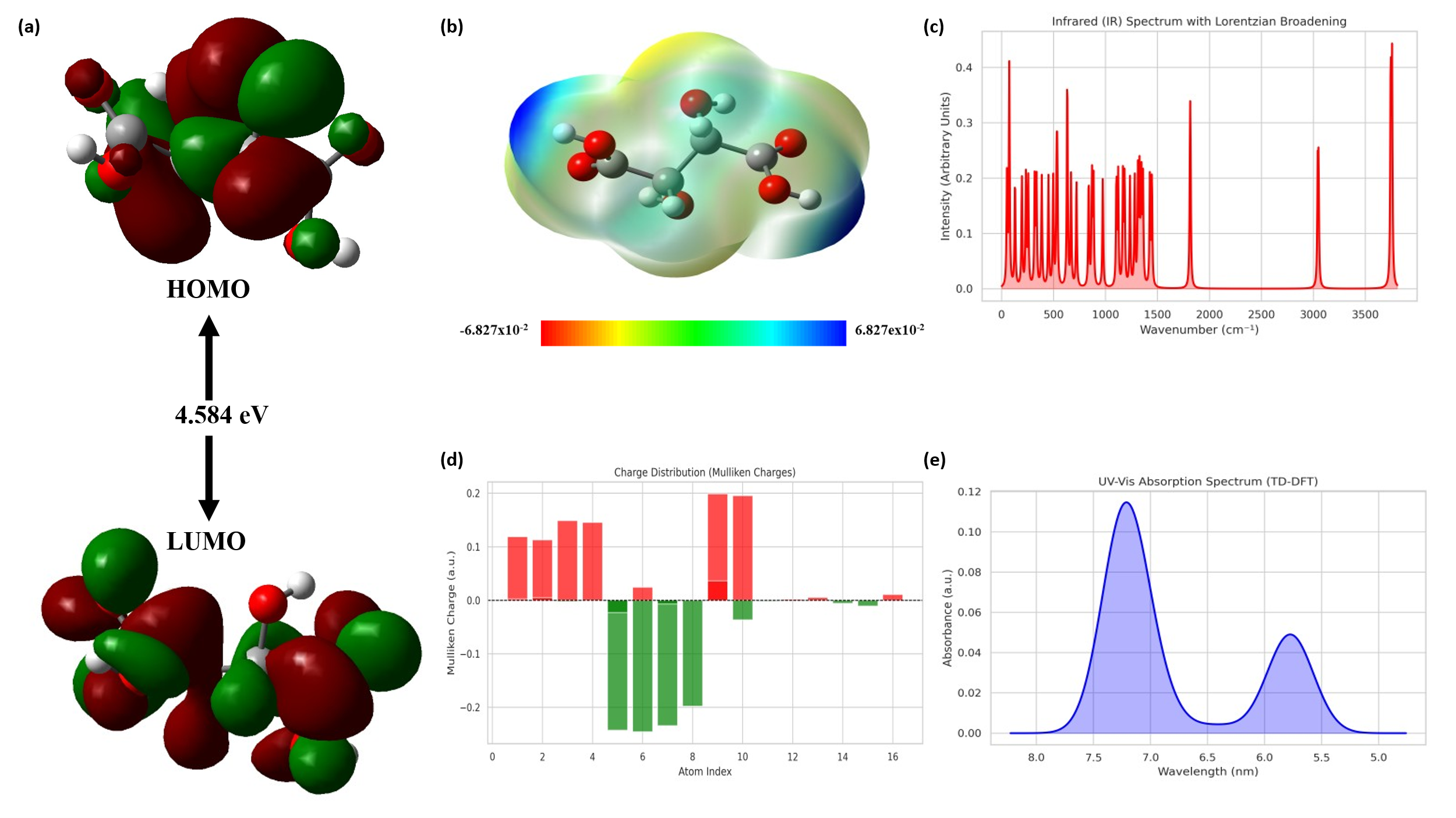
**Supplementary Figure S4:** DFT analysis for compound 3999 (d-Tartaric acid) **(a)**HOMO-LUMO orbitals showing a 4.584 eV energy gap **(b)** electrostatic potential map of compound 3999 (d-Tartaric acid), **(c)** Simulated IR spectrum confirms vibrational modes with no imaginary frequencies, supporting structural stability **(d)** Mulliken charge distribution highlights nucleophilic (green) and electrophilic (red) atomic centres **(e)** UV-Vis absorption spectrum shows a strong peak near 6.0 nm, indicating electronic transition activity
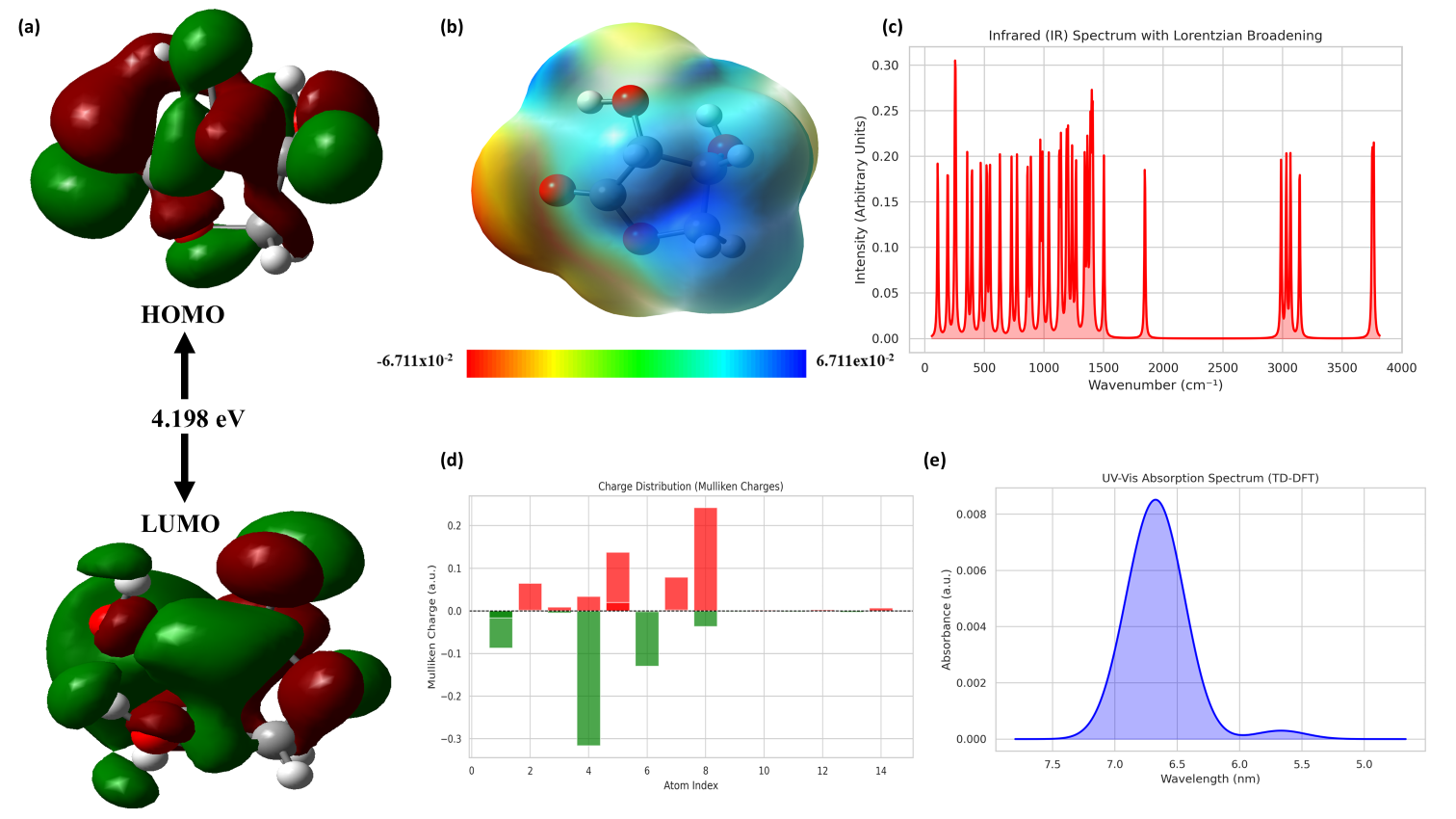
**Supplementary Figure S5:** DFT analysis for compound 4703 (D-Erythronolactone) **(a)** HOMO-LUMO orbitals showing a 4.198 eV energy gap **(b)** electrostatic potential map of the compound **(c)** Simulated IR spectrum confirms vibrational modes with no imaginary frequencies, supporting structural stability **(d)** Mulliken charge distribution highlights nucleophilic (green) and electrophilic (red) atomic centres **(e)** UV-Vis absorption spectrum shows a strong peak near 6.0 nm, indicating electronic transition activity
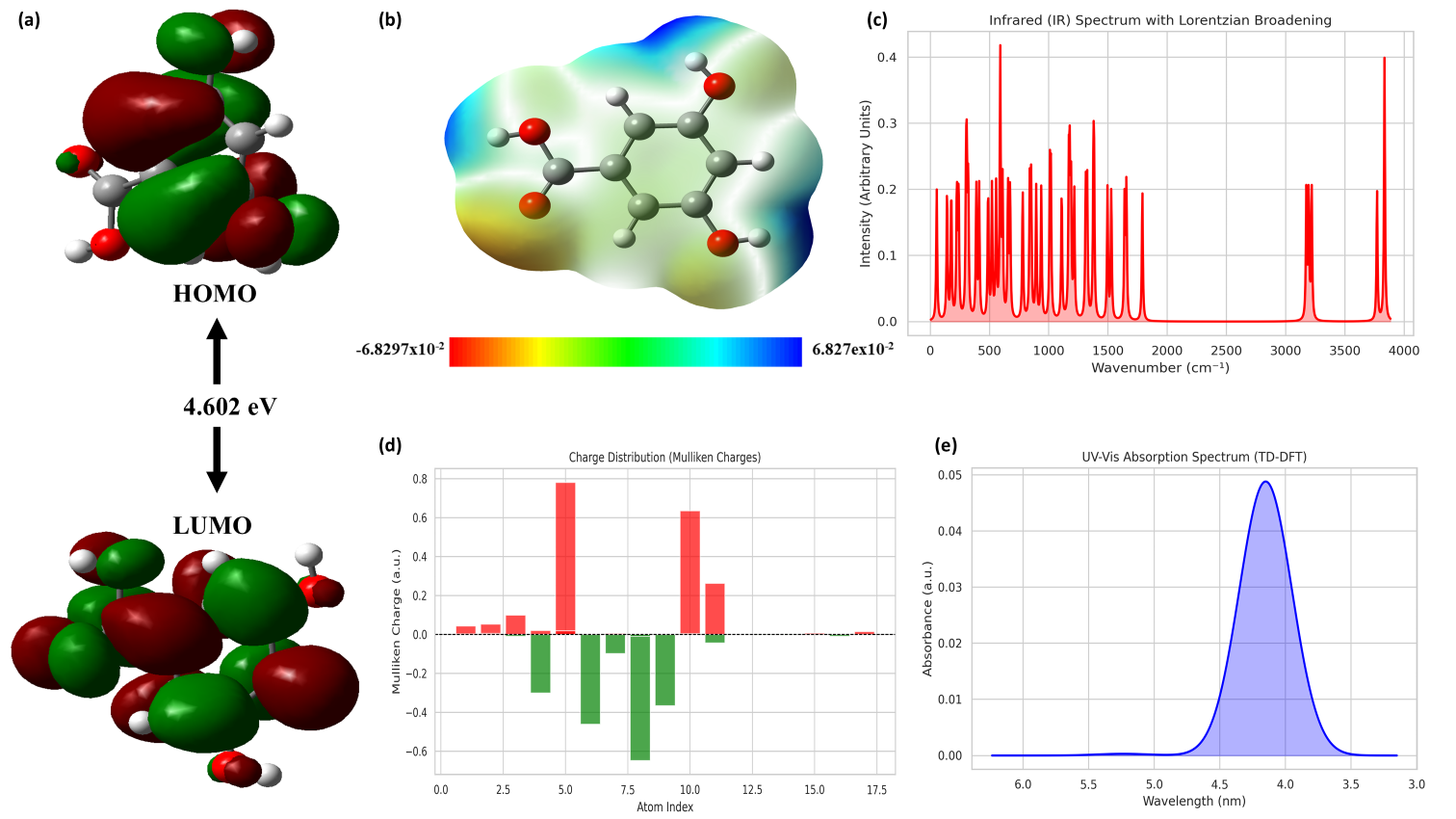
**Supplementary Figure S6:** DFT analysis for compound 5991 (3,5-Dihydroxybenzoic acid) **(a)** HOMO-LUMO orbitals showing a 4.602 eV energy gap **(b)** electrostatic potential map of the compound **(c)** Simulated IR spectrum confirms vibrational modes with no imaginary frequencies, supporting structural stability **(d)** Mulliken charge distribution highlights nucleophilic (green) and electrophilic (red) atomic centres **(e)** UV-Vis absorption spectrum shows a strong peak near 6.0 nm, indicating electronic transition activity


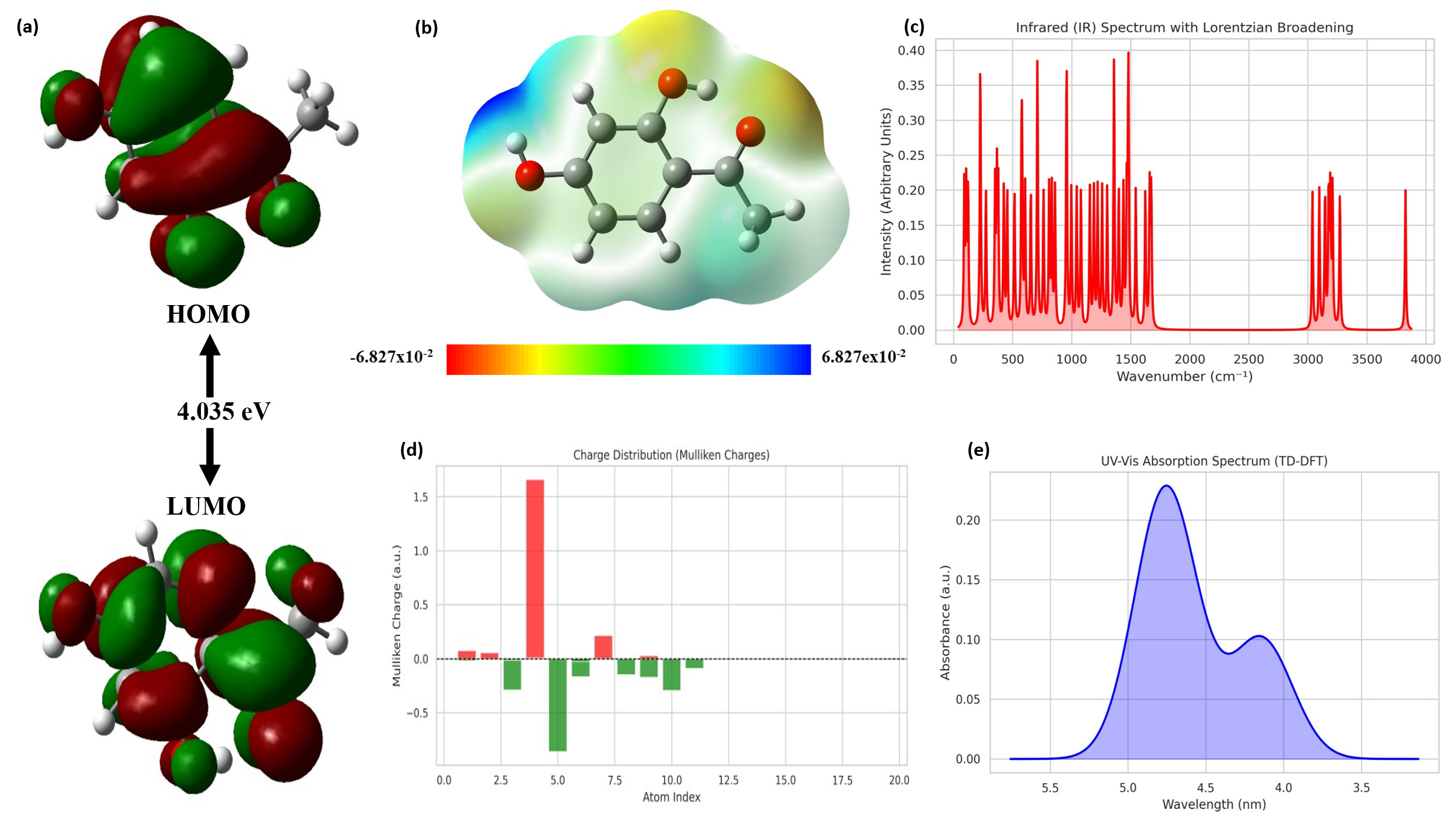
**Supplementary Figure S7:** DFT analysis for compound 6552 (2',4'-Dihydroxyacetophenone) **(a)** HOMO-LUMO orbitals showing a 4.0.35 eV energy gap **(b)** electrostatic potential map of the compound **(c)** Simulated IR spectrum confirms vibrational modes with no imaginary frequencies, supporting structural stability **(d)** Mulliken charge distribution highlights nucleophilic (green) and electrophilic (red) atomic centres **(e)** UV-Vis absorption spectrum shows a strong peak near 6.0 nm, indicating electronic transition activity


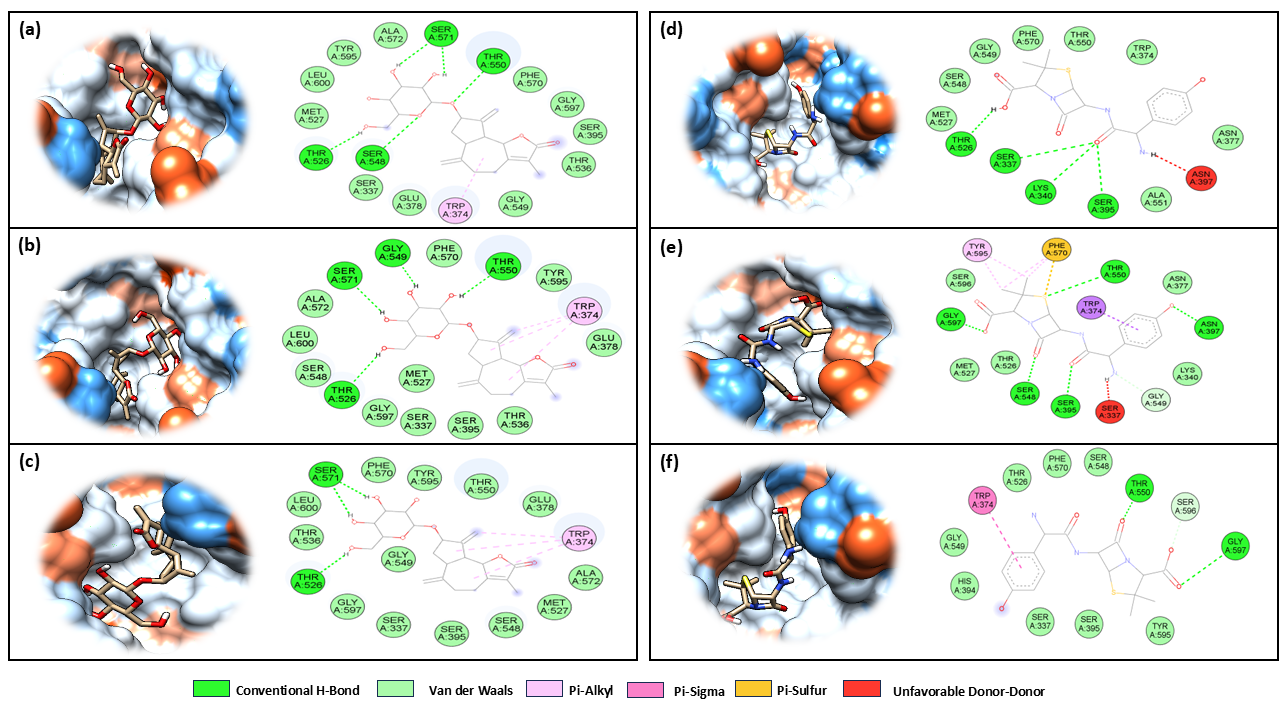


**Supplementary Figure S8:** Intermolecular interaction analysis for KSG motif **(a)** Interaction between wild-type and Glucozaluzanin C **(b)** Interaction between mutant K547G and Glucozaluzanin C **(c)** Interaction between mutant K547T and Glucozaluzanin C **(d)** Interaction between wild-type and Amoxicillin **(e)** Interaction between mutant K547G and Amoxicillin **(f)** Interaction between mutant K547T and Amoxicillin


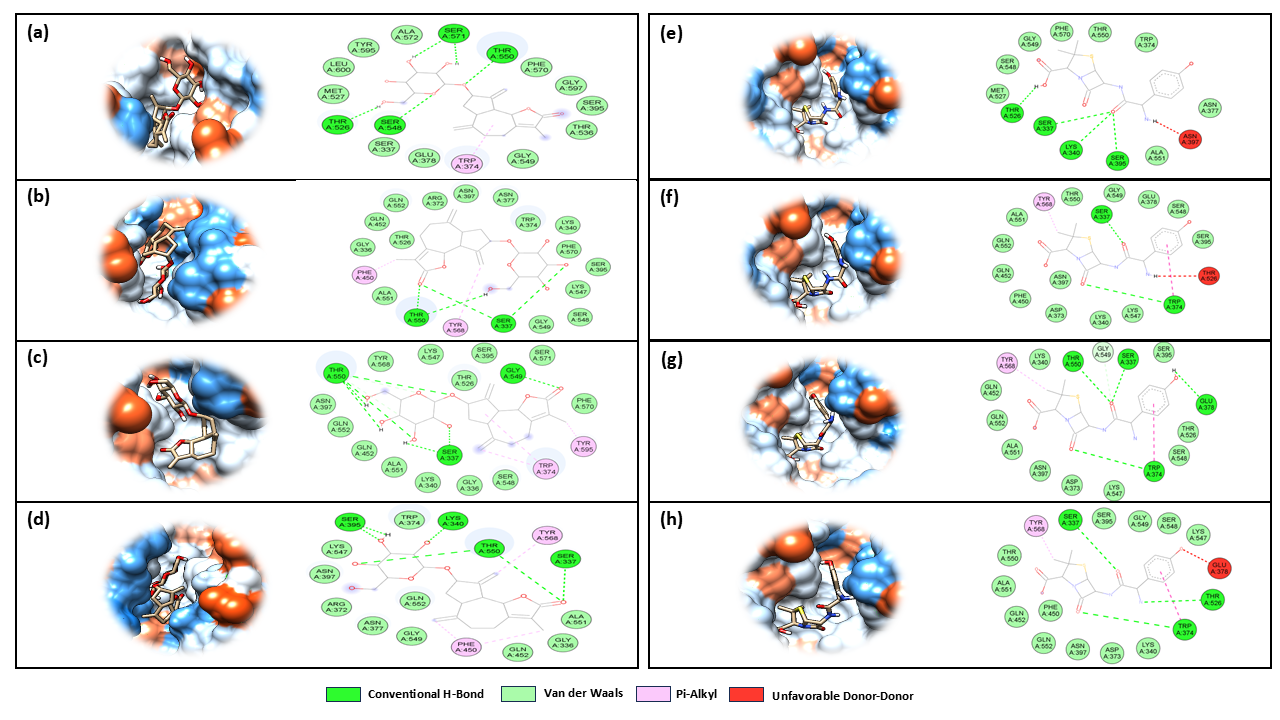


**Supplementary Figure S9:** Intermolecular interaction analysis for STMK motif **(a)** Interaction between wild-type and Glucozaluzanin C **(b)** Interaction between mutant T338A and Glucozaluzanin C **(c)** Interaction between mutant T338G and Glucozaluzanin C **(d)** Interaction between mutant T338P and Glucozaluzanin C **(e)** Interaction between wild-type and Amoxicillin **(f)** Interaction between mutant T338A and Amoxicillin **(g)** Interaction between mutant T338G and Amoxicillin **(h)** Interaction between mutant T338P and Amoxicillin


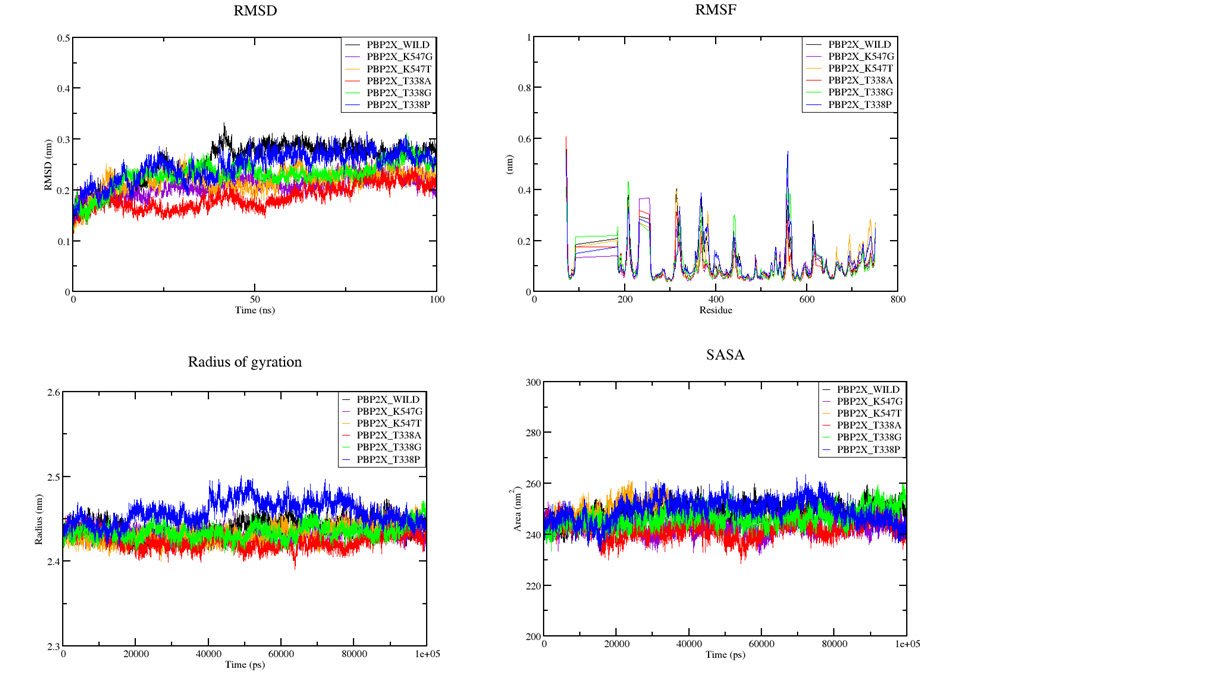


**(b)**

**(a)**

**(d)**

**(c)**

**Supplementary Figure S10:**  Molecular Dynamics (MD) simulations of the apo form of PBP2x wild-type (black) and mutant variants K547G (violet), K547T (orange), T338A (red), T338G (green), and T338P (blue). The MD plots represent: (a) RMSD to assess overall structural stability; (b) RMSF for residue-wise flexibility; (c) Rg indicating compactness; (d) SASA reflecting protein surface exposure during the simulation.


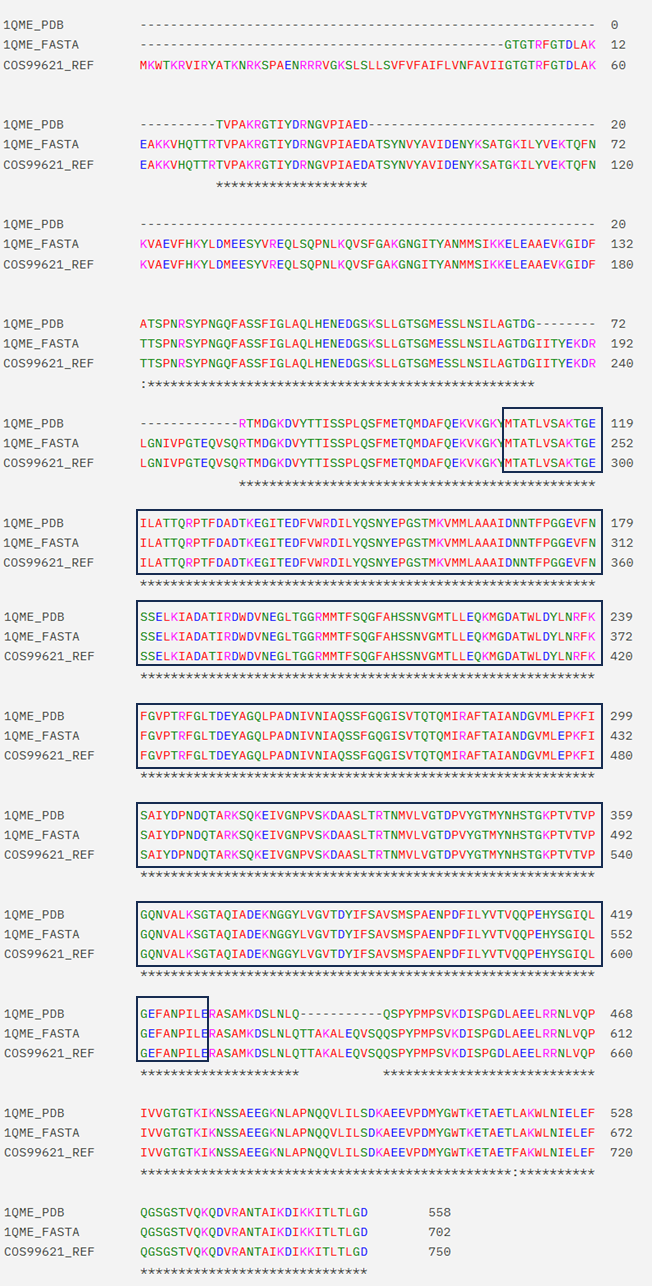


**Supplementary Figure S11:** Sequence alignment of the crystal structure (1QME_PDB), translated FASTA sequence (1QME_FASTA), and reference protein (COS99621_REF) of *Streptococcus pneumoniae* PBP2x. Missing residues in the PDB structure (indicated by dashes) correspond to unresolved region


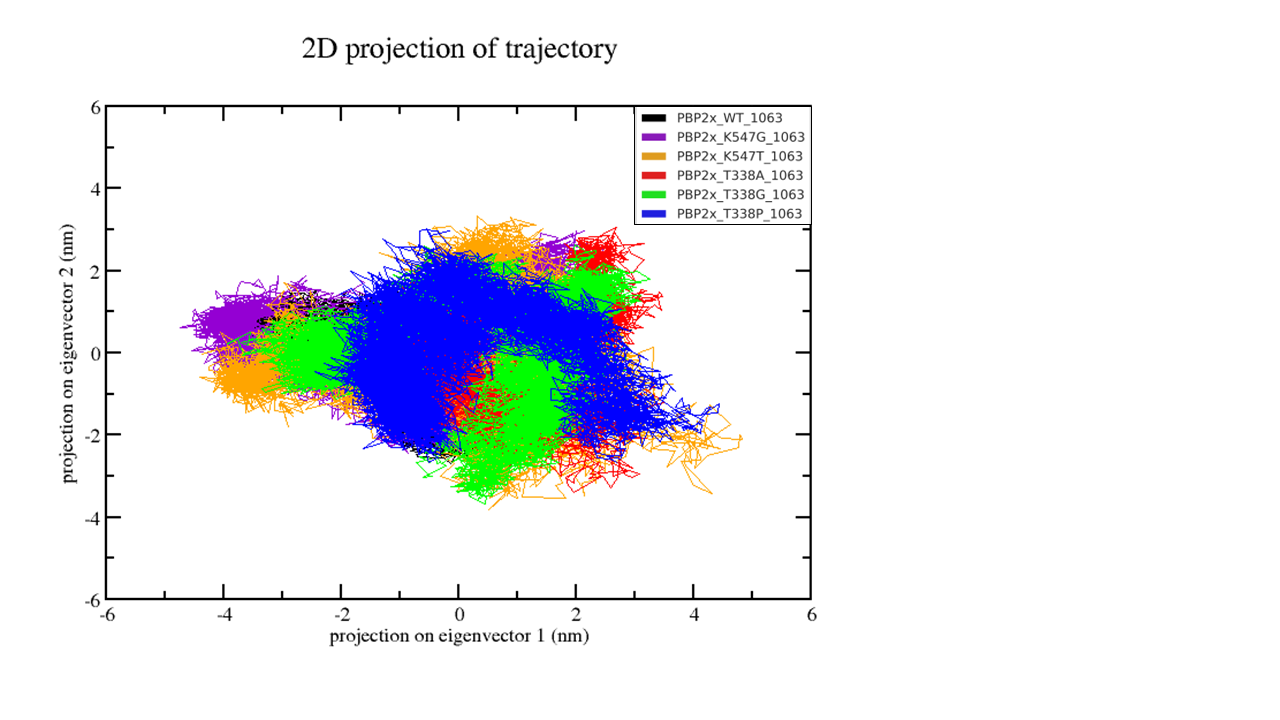


**Supplementary Figure S12:** PCA plot showing the projection of MD trajectories of PBP2x wild-type and mutant complexes with Glucozaluzanin C (1063) onto the first two eigenvectors. Distinct clustering patterns reflect the conformational variability among the variants.


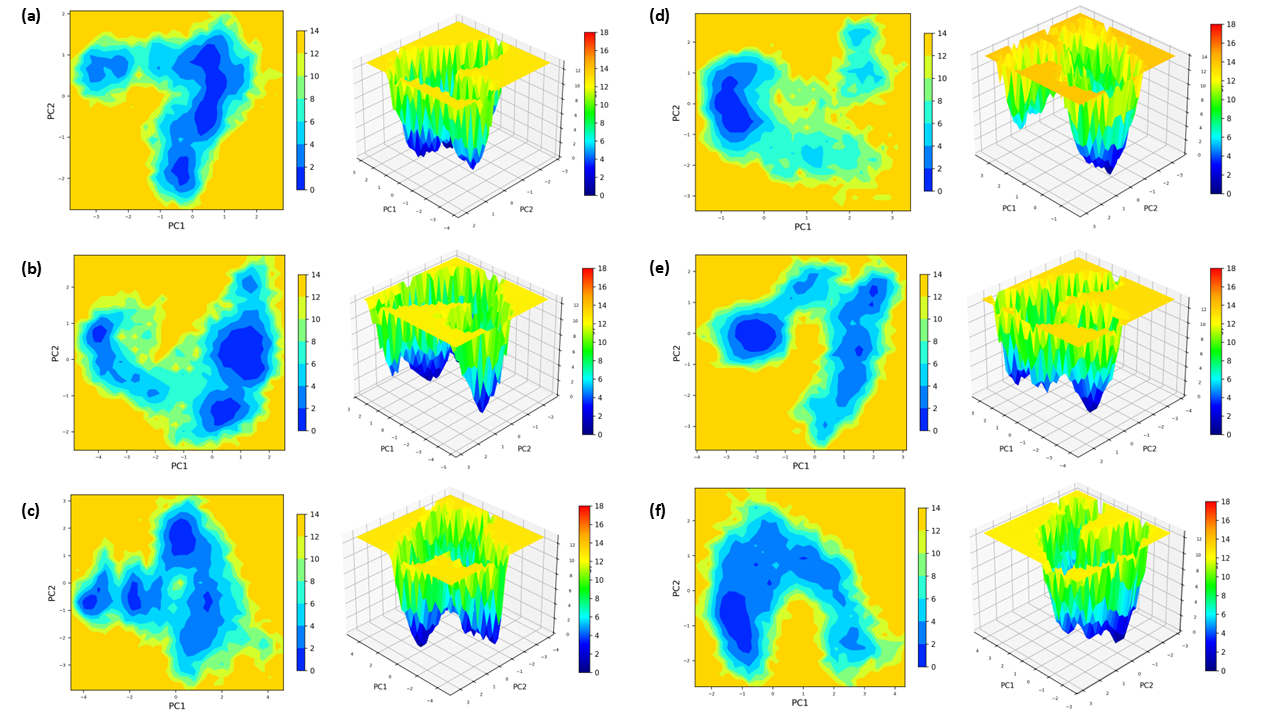
**Supplementary Figure S13:** FEL plot 2D and 3D plots **(a)** Wild type and Glucozaluzanin C complex **(b)** Mutant K547G and Glucozaluzanin C complex **(c)** Mutant K547T and Glucozaluzanin C complex **(d)** Mutant T338A and Glucozaluzanin C complex **(e)** Mutant T338G and Glucozaluzanin C complex **(f)** Mutant T338P and Glucozaluzanin C complex


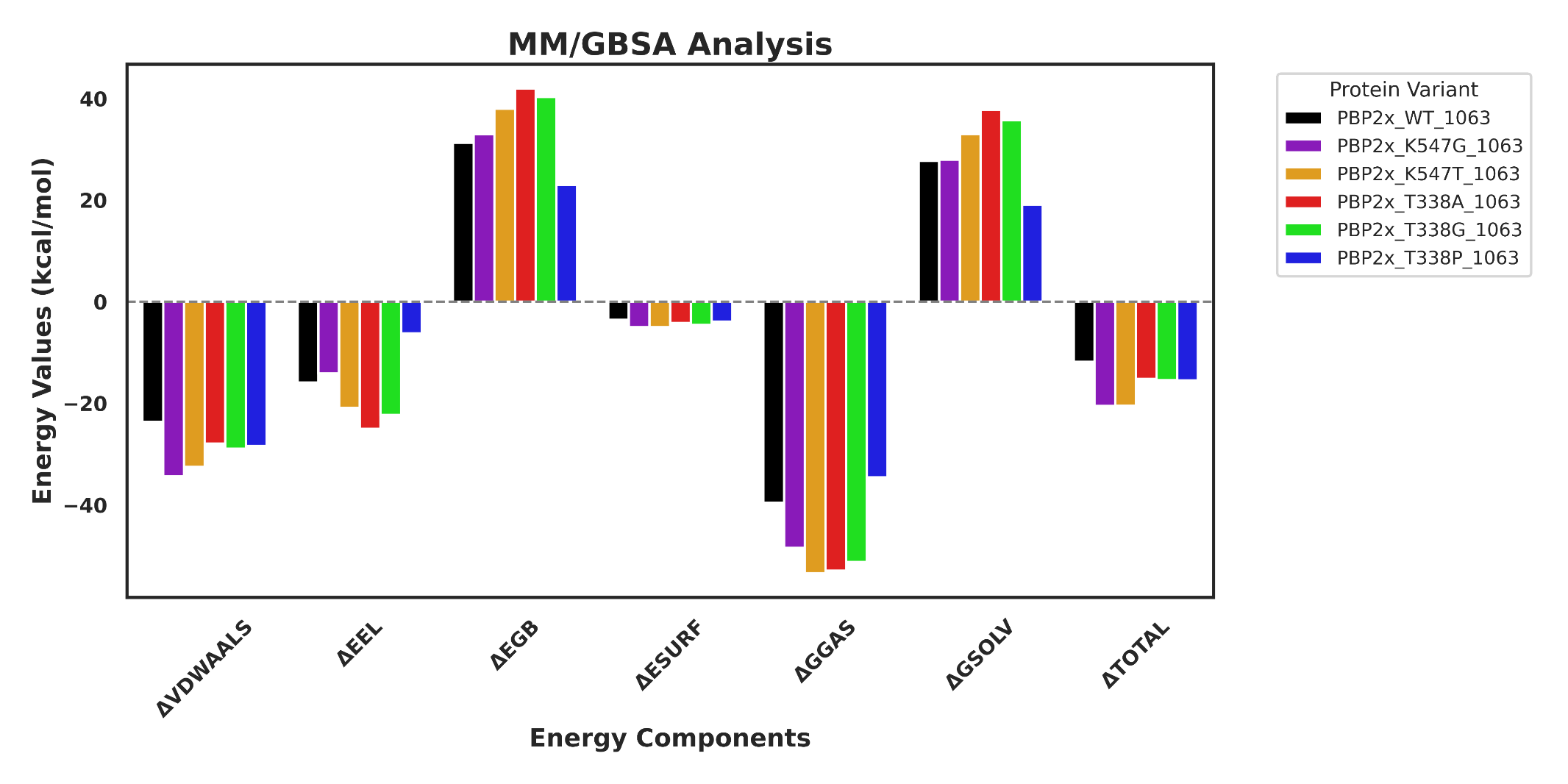
**Supplementary Figure S14:** MM/GBSA energy component comparison for Glucozaluzanin C binding to PBP2x wild-type and mutants. Bars represent contributions from van der Waals, electrostatic, solvation, and total binding energies across all variants.


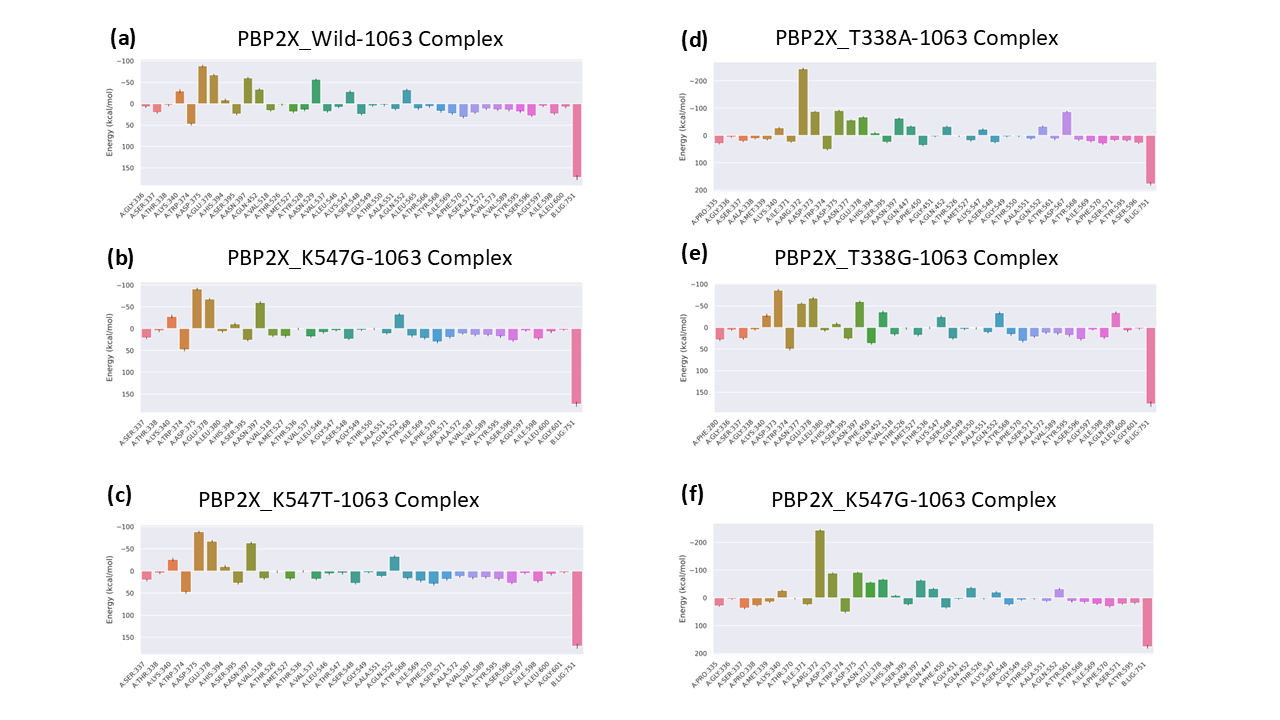
**Supplementary Figure S15:** Per-residue energy decomposition analysis of Glucozaluzanin C bound to PBP2x wild-type and mutant complexes. Panels (a–f) display the energy contributions of individual residues in each complex.
